# Spatial host–microbiome sequencing reveals niches in the mouse gut

**DOI:** 10.1038/s41587-023-01988-1

**Published:** 2023-11-20

**Authors:** Britta Lötstedt, Martin Stražar, Ramnik Xavier, Aviv Regev, Sanja Vickovic

**Affiliations:** 1 Klarman Cell Observatory, Broad Institute of MIT and Harvard, Cambridge, MA USA; 2 Science for Life Laboratory, Department of Gene Technology, KTH Royal Institute of Technology, Stockholm, Sweden; 3 New York Genome Center, New York, NY USA; 4 Broad Institute of MIT and Harvard, Cambridge, MA USA; 5 Center for Microbiome Informatics and Therapeutics, Massachusetts Institute of Technology, Cambridge, MA USA; 6 Department of Molecular Biology, Center for Computational and Integrative Biology, Massachusetts, General Hospital, Harvard Medical School, Boston, MA USA; 7 Department of Biology, Massachusetts Institute of Technology, Cambridge, MA USA; 8 Department of Biomedical Engineering and Herbert Irving Institute for Cancer Dynamics, Columbia University, New York, NY USA; 9 Science for Life Laboratory, Department of Immunology, Genetics and Pathology, Beijer Laboratory for Gene and Neuro Research, Uppsala University, Uppsala, Sweden; 10 Present Address: Genentech, South San Francisco, CA USA

**Keywords:** Next-generation sequencing, Genomic analysis

## Abstract

Mucosal and barrier tissues, such as the gut, lung or skin, are composed of a complex network of cells and microbes forming a tight niche that prevents pathogen colonization and supports host–microbiome symbiosis. Characterizing these networks at high molecular and cellular resolution is crucial for understanding homeostasis and disease. Here we present spatial host–microbiome sequencing (SHM-seq), an all-sequencing-based approach that captures tissue histology, polyadenylated RNAs and bacterial 16S sequences directly from a tissue by modifying spatially barcoded glass surfaces to enable simultaneous capture of host transcripts and hypervariable regions of the 16S bacterial ribosomal RNA. We applied our approach to the mouse gut as a model system, used a deep learning approach for data mapping and detected spatial niches defined by cellular composition and microbial geography. We show that subpopulations of gut cells express specific gene programs in different microenvironments characteristic of regional commensal bacteria and impact host–bacteria interactions. SHM-seq should enhance the study of native host–microbe interactions in health and disease.

## Abstract (web summary)

Spatial host–microbiome sequencing simultaneously profiles microbes and host transcriptomes from mouse colons.

## Main

Mucosal and barrier tissues are ecosystems of multiple host cell types and a complex microbiome that vary in space and time. Antigen recognition and innate immune responses^1^ in the host, and molecular mechanisms derived from the microbiome, together prevent pathogen colonization and support the establishment of the host–microbiome spatial niche and host–microbial symbiosis^2^. Conversely, spatial dysregulation^3,4^ in diseases such as inflammatory bowel disease (IBD) can lead to dysfunction of the gut barrier^5,6^.

Characterizing and understanding the host–microbiome spatial niche requires detailed measurement of the identity and molecular characteristics of host cells and microbiome species and their interrelations in a spatial context. On the microbiome side, spatial metagenomics methods^7^ are emerging to map bacteria by either imaging^8,9^ or metagenomic plot sampling^10^. However, such studies focused on smaller regions, such as inter-fold, mucosal or lumen regions in the gut, and typically used broad taxonomy assignments, reaching family level at best^10,11^, with few reports at the level of specific genera or species^9,12–14^. Moreover, metagenomic plot sampling, so far the only approach for spatial bacterial sequencing in situ^10^, does not currently profile host gene expression. On the host side, single-cell genomics, including single-cell RNA sequencing (scRNA-seq), has been instrumental to characterize the cellular composition of tissues, for the host^15^, resident microbes^16,17^ or joint profiling of host and viral amplicon sequences^18^, but without spatial information. Spatial transcriptomics methods, either imaging based or sequencing based, enable cell type mapping in situ^19–24^ but have not yet been applied to simultaneously profile both host and microbiome in a spatial context.

**Fig. 1:**
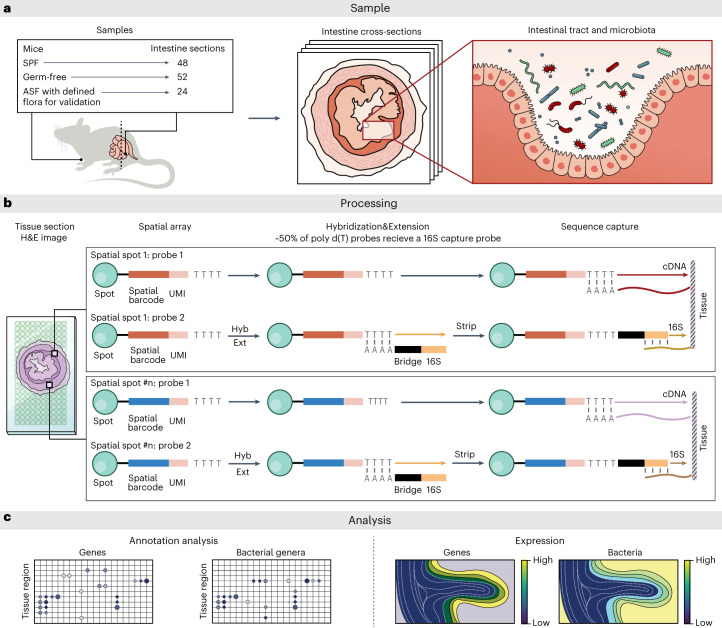
SHM-seq. **a**, Three different mouse conditions used in the study analyzing cross-sections in the mouse gut. **b**, Tissue sections from mouse colons were placed on a barcoded glass array, with a barcoded surface adapted for simultaneous capture of polyadenylated host transcripts and 16S bacterial rRNA. Tissue sections were imaged, cells were permeabilized and cDNA was synthesized on the array surface before library preparation and sequencing. **c**, Data analysis identifies regional gene programs, their cell type constituents, association with mouse condition and regional association with specific commensal bacteria. Hyb, hybridization; Ext, extension.

In this study, we bridged this gap by developing spatial host–microbiome sequencing (SHM-seq; Fig. 1), a robust all-sequencing-based technology that leverages previous advancements in spatial transcriptomics^25,26^ and provides histology, spatial RNA-seq and spatial 16S sequencing using readily available instrumentation to profile the host’s expression responses in relation to microbial biogeography. We applied it in the model system of the mouse colon and show here a roadmap for interrogating spatial gene expression programs in correlation with bacterial presence.

## Results

### SMH-seq

We developed SHM-seq by adapting spatial transcriptomics^25^, where mRNA is captured by probes on a glass slide followed by profiling, to enable simultaneous capture of polyadenylated (host) transcripts and hypervariable (V4) regions of the 16S ribosomal RNA (rRNA) (Methods and Fig. 1). Specifically, we first produced solid-phase spatial transcriptomics slides covered with uniquely barcoded and spatially addressable poly(d)T capture probes^25–27^, with ~1,000 distinct DNA features (that is, spatial spots) deposited and covalently linked to a glass substrate (Methods). We enzymatically modified these spatially barcoded features on the glass array to enable simultaneous capture of polyadenylated transcripts (~50% surface capture probes) and hypervariable (V4) regions of the 16S rRNA^28^ (~50% surface capture probes) through a hybridization and extension reaction (Methods, Fig. 1 and Supplementary Fig. 1). Next, we placed frozen tissue sections on the optimized glass surface, stained them with hematoxylin and eosin (H&E) and imaged the tissue histology by bright-field microscopy. Finally, after imaging, we permeabilized the cells, allowing capture of host polyadenylated transcripts and bacterial 16S sequences on the array. The result was direct spatial DNA barcoding of host transcripts and bacterial species, which were sequenced by Illumina sequencing^26^.

To test SHM-seq, we applied it to profile intestinal cross-sections from the colon of C57BL/6 mice grown under typical conditions (specific pathogen free (SPF)) or as germ free (GF) or altered Schaedler flora (ASF) mice (Fig. 1 and Methods). GF mice provide a negative control; ASF mice, which contain only a defined floral community, provide a clear target for validation of the capture of expected bacterial species; and regular C57BL/6 SPF mice represent a complex case study with unaltered gut flora. In total, we applied SHM-seq to 124 tissue sections and collected data from 15,321 spatial spots (covered by the tissue) across the three conditions (Supplementary Tables 1 and 2 and Supplementary Fig. 2).

### A deep learning approach for taxonomy classification

**Fig. 2:**
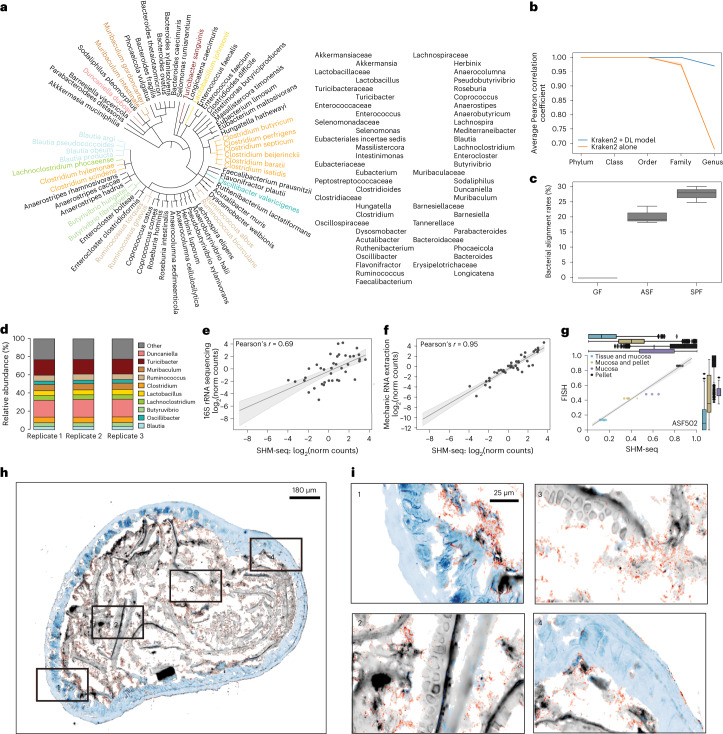
SHM-seq accurately captures bacterial representation and abundances in SPF and ASF mice. **a**, Bacterial reference of the mouse gut microbiome. Phylogenetic tree of SPF colonic content, representing the 65 species colored to highlight taxonomic families and genera. **b**, Enhanced annotation performance of the deep learning model. Average Pearson correlation coefficient (*y* axis) between true and predicted taxonomic labels from spatial spots (Methods) on five taxonomic levels (*x* axis) when using Kraken2 (orange) or Kraken2 together with the deep learning model (blue) (*y* axis) (*n* = 3). Error bars: 95% confidence intervals. **c**, Highly specific mapping of bacterial reads. Overall bacterial alignment rates to reference genomes (*y* axis, %) for GF (left, *n* = 3), ASF (middle, *n* = 3) and SPF (right, *n* = 3) tissue sections using spatial 16S sequencing. **d**, High reproducibility of bacterial abundances in SPF mouse colons by SHM-seq. Percentage (*y* axis) of the top 10 most abundant bacteria genera in each of three independent samples of SPF mouse colons (*x* axis). **e**, SHM-seq compares well to 16S rRNA sequencing. Pseudo-bulk abundances of bacterial genera (dot) from SHM-seq (*x* axis, SPF mice, *n* = 3) and bulk 16S rRNA sequencing^62^ (*y* axis, SPF mice, *n* = 3). Top left: Pearson *r*. **f**, Enzymatic (SHM-seq) extraction of bacterial content agrees with established mechanic extraction. Pseudo-bulk abundances of each bacterial genera (dot) from SHM-seq (*x* axis, SPF mice, *n* = 3) and mechanical extraction (*y* axis, SPF mice, *n* = 3). **g**–**i**, SHM-seq agreement with FISH probes targeting ASF502. **g**, Distribution (box plot, normalized signals per region) and individual measurements (scatter plot, mean signal per region and sample, *n* = 6) of ASF502 counts by FISH (*y* axis) and SHM-seq (*x* axis). Shaded areas: 95% confidence interval. **c**,**g**, Box plots: center black line, median; color-coded box, interquartile range; error bars, 1.5× interquartile range; black dots; outliers. **h**, Cross-section of an ASF mouse colon (scale bar, 180 μm) with four regions (red rectangles) and their zoom-ins (**i**) (scale bar, 25 μm). Colors: tissue (blue), fibers (gray) and ASF502 (red). **e**,**f**, Line: linear regression model fit. Shaded areas: 95% confidence interval. norm, normalized; DL, deep learning.

Although, for spatial transcriptomics (host) data, we used an established processing pipeline^29^ (Methods), we devised a novel taxonomy assignment pipeline to process the spatial microbiome data. First, we created a custom, gold standard bacterial genome reference for our experiment (Methods), based on species detected in dedicated shotgun metagenomic sequencing data, comprising 65 most abundant species from 39 genera present in our bulk reference samples in at least 0.1% abundance, a cutoff chosen as offering the most accurate mapping metrics (Fig. 2a, Supplementary Fig. 3a and Methods). Next, we compared the performance in terms of taxonomic assignment (by Kraken2 (ref.^30^)) when using this custom reference versus using other bacterial reference databases, including the National Center for Biotechnology Informationʼs (NCBI) RefSeq^31^ whole genome and NCBI’s 16S rRNA databases (Methods and Supplementary Fig. 3b–d). Although our customized gold standard (restricted) whole genome reference (SPF: 65 species; ASF: eight species) had higher mapping accuracy and lower false-positive rate on both real and simulated SHM-seq data, the RefSeq references performed reasonably as well, making them a viable option when dedicated metagenomics data cannot be collected for a customized reference. Simulated data additionally showed that the database type (whole genome versus 16S rRNA), size and sequencing read length all impact the performance of taxonomic assignments (Supplementary Fig. 3e).

We next devised a taxonomy assignment approach, where spatially captured sequences were first classified using Kraken2 (ref.^30^) (Methods), and those without a taxonomic classification were then processed by a novel deep learning approach (Methods). Our deep learning model (Supplementary Fig. 4a) is based on convolutional and recurrent neural networks, which process a read from both directions, seek local sequence patterns and their distant interactions and are trained to predict the most likely taxonomic assignment (Methods). We assessed its performance using simulated bacterial reads with attached taxa labels, mimicking data otherwise obtained with SHM-seq (Methods).

The deep learning model enhanced performance compared to using Kraken2 (Fig. 2b and Supplementary Fig. 4b,c) or QIIME 2 (ref.^32^), a commonly used 16S rRNA analysis tool (average Pearson *r*: 0.97 (Kraken2 + deep learning model) and 0.21 (QIIME 2), *P* ≤ 10^−4^, average Bray–Curtis dissimilarity: 0.06 and 0.46, respectively, genus level; Supplementary Fig. 4d). First, the deep learning model (used alone) assigned sequences to genera with 97% accuracy on a test dataset of 20% of the data. Next, we used the simulated data to assess taxonomic assignment metrics by comparing the predicted to the true taxonomic labels for data unseen by the model during training. The deep learning model after Kraken2 significantly outperformed Kraken2 alone on genus-level assignment, by several measures, including (1) higher similarity between relative bacterial abundances based on the model’s assignment versus the ground truth (average Pearson *r* for Kraken2 + deep learning model versus Kraken2 alone: 0.97 versus 0.68, *P* ≤ 10^−4^; Fig. 2b); (2) higher similarity in bacterial composition, evaluated at a resolution of 1,000 randomly assigned spatial spots (average Bray–Curtis dissimilarity 0.06 versus 0.15; Supplementary Fig. 4b); and (3) higher total accuracy (92% versus 84%, *P* ≤ 10^−4^), higher F1 score (89% versus 85%, *P* ≤ 10^−4^) and lower false-positive rate (8% versus 16%, *P* ≤ 10^−4^; Supplementary Fig. 4c), when evaluated as bulk-like samples. Thus, the deep learning model can improve the taxonomic assignments in SHM-seq data.

### Sensitive and specific bacterial rRNA and host mRNA capture

We evaluated SHM-seq by (1) specificity and sensitivity of bacterial capture rates in SHM-seq compared to bulk 16S rRNA sequencing and by fluorescence in situ hybridization (FISH); and (2) host RNA-seq quality metrics obtained by SHM-seq compared to spatial transcriptomics alone.

To assess specificity (the fraction of sequencing reads mapping to genomic regions in the gold standard reference) and sensitivity (the fraction of expected species detected with SHM-seq), we analyzed profiles from the defined community in ASF^33^ mice as a positive control and from GF mice as a negative control (Fig. 1 and Methods). On average, 22% of all reads in ASF mice samples (*n* = 3) aligned to the bacterial reference, whereas only 0.008036% of reads from GF mice samples were assigned to any of the 65 species in the reference (*n* = 3) (Fig. 2c). For ASF samples, bacterial reads mapped to the expected locations in the respective ASF reference genomes highlighting the specificity of our targeted capture (mean reads in expected genomic bin: 97.0 ± 1.5% s.e.m., *n* = 18 tissue sections; Supplementary Fig. 5), with most reads (85.7 ± 4.5% mean ± s.e.m., *n* = 18 tissue sections) mapping on average to the expected capture region of the 16S rRNA gene (Supplementary Fig. 6). Highlighting the sensitivity of SHM-seq, all of the expected bacterial species were captured in the ASF samples, with ASF519 and ASF502 as the dominating bacteria (Supplementary Fig. 7a), in line with previous bulk RT–qPCR results^34^ (Pearson *r* = 0.85; Supplementary Fig. 7b) and with high reproducibility across replicates (Supplementary Fig. 7a). SHM-seq even detected ASF360, which was previously reported to be difficult to detect at low abundance using RT–qPCR^35^.

As a more complex case study, we further tested SHM-seq’s performance in bacterial capture in SPF mice (Methods; *n* = 3). On average, 28% of all reads aligned to the bacterial genome reference (Fig. 2c) and were assigned to 39 genera in our metagenomic reference (22 of which were present at >1% abundance), with *Duncaniella, Turicibacter* and *Muribaculum* the most abundant (Fig. 2d). The genera detected and their relative abundances correlated well with 16S rRNA sequencing (Pearson *r* = 0.69, *P* ≤ 10^−4^; Fig. 2e), and, on average, 90.7 ± 1.7% (mean ± s.e.m.) of SHM-seq reads (*n* = 9 tissue sections) mapped to the expected 16S rRNA capture region. Notably, our enzymatic cell permeabilization protocol was as efficient for preparing (bulk) bacterial samples as was traditional mechanical extraction of nucleic acids (Pearson *r* = 0.95, *P* ≤ 10^−4^; Fig. 2f).

To further validate the specificity of spatial capture of bacterial genomes in different regions of interest, we compared the bacterial abundance profiles obtained with SHM-seq in ASF mice with those measured by FISH (Methods) with five fluorescent bacterial detection probes: a positive control to detect all bacterial species, probes targeting three distinct ASF species and a negative control. We detected and quantified the fluorescence signal over three major tissue regions (Methods and Supplementary Fig. 8a–d). The abundances of the overall positive control and of each of the three ASF-specific bacterial species in FISH correlated significantly with the SHM-seq measurements (average Spearman ρ; ASF502: 0.72, ASF360: 0.72, ASF519: 0.55, positive control: 0.75, *P* ≤ 10^−4^; Fig. 2g–i and Supplementary Fig. 8e–g).

Host RNA-seq quality metrics were similar between SHM-seq and spatial transcriptomics. There were no significant differences in RNA-seq read mapping rates or unique molecular identifier (UMI) counts between spatial transcriptomics and SHM-seq in either SPF or ASF mice (*n* = 3; Supplementary Fig. 9a–d): 66% and 63% of the spatially captured reads were uniquely mapped, and pseudo-bulk UMI counts correlated highly (Pearson *r* = 0.95 and 0.92, respectively). Furthermore, there was high agreement in host expression profiles when we used regular spatial transcriptomics arrays (only poly(d)T capture) with the permeabilization method developed solely for disrupting host cells versus the method used for disrupting both host and bacterial cells (Pearson *r* = 0.94; Supplementary Fig. 9e,f). Thus, the surface treatment, permeabilization method and library preparation used in SHM-seq compare in specificity and sensitivity to commonly used methods for accessing bacterial sample composition and for spatial host expression profiling.

### Defining spatial patterns of bacterial and host expression

**Fig. 3:**
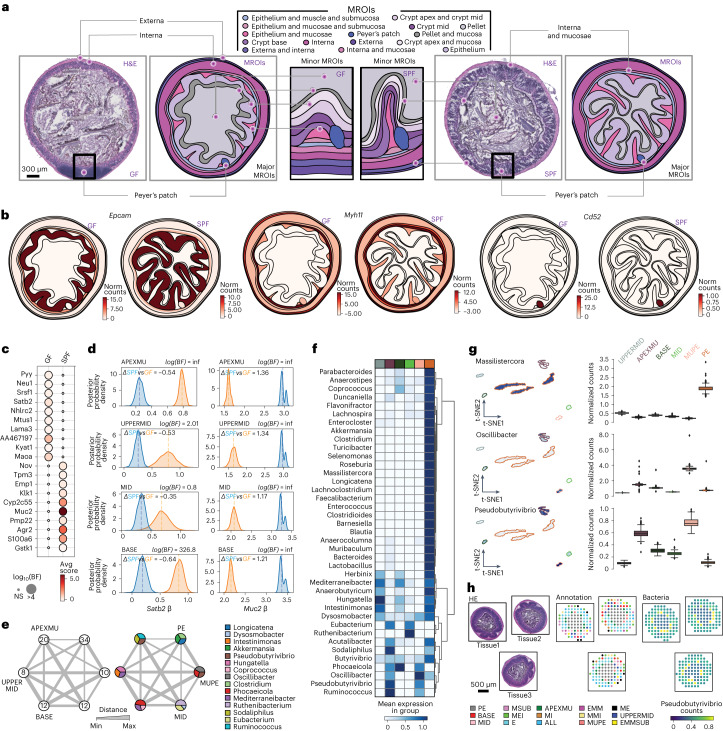
Spatial detection of bacteria and host gene expression with SHM-seq. **a**, MROI in the mouse colon. H&E-stained tissue sections from GF (left) and SFP (right) mice (left panels) annotated and visualized with vector representations (right panels), showing bacteria and host expression in major and minor MROIs associated with each anatomical tissue layer (right panels). Scale bar, 300 μm. **b**, Spatial host gene expression in three major MROIs. Expression (color bar, normalized gene expression) of selected spatially variable genes in GF (left) and SPF (right) tissue sections in major MROIs. **c**, Differential gene expression between mouse conditions. Significance (dot size, log_10_BF; Methods) of differential expression and expression level (normalized gene expression) of the top 10 genes (rows) differentially expressed between GF and SPF mouse tissue sections (columns) (Methods). **d**, Gene expression differences between morphological regions. Posterior distributions of the region-specific coefficient parameters (β) of *Satb2* (left) and *Muc2* (right) in four MROIs describing colonic crypts in SPF (blue) and GF (orange) mice. Dashed lines: mean of each distribution. **e**, Bacteria detected across six MROIs in SPF mouse tissues. Number of (left) and top three most abundant (right) bacteria genera (color code) detected in minor MROIs. Line thickness: average Euclidean distances between MROIs. **f**, Regional abundance of taxa. Scaled normalized bacterial counts (normalized counts scaled within each genus, color bar) in MROIs (color code, columns) for each detected bacteria (rows). **g**, Association between taxa and spatial regions. t-distributed stochastic neighbor embedding (t-SNE) of scaled bacterial count profile of each spatial spot (color scale, dots, *n* = 4,655, left panel) and the distribution of normalized bacterial count for all spatial spots (right panel) in six minor MROIs (color code) in SPF mice for different genera. Boxplots: Center black line, median; color-coded box, interquartile range; error bars, 1.5x interquartile range; black dots; outliers. **h**, Reproducibility of bacterial associations across individual sections. H&E (left), MROI annotations (color code, middle) and normalized bacterial count for *Pseudobutyrivibrio* (color scale, right) in three tissue sections. Circles: spatial spots. Scale bar, 500 μm. Abbreviations as in Methods (**c**–**h**). MROI color code shared (**f**,**g**). Norm, normalized; NS, not significant; inf, infinity.

To recover the spatial organization of microbes and host from our data, we defined the expression of host genes and abundance of bacterial genera in each spot, mapped those to 16 defined morphological regions of interest (MROIs) (Fig. 3a) to identify characteristic patterns and, finally, visualized our data as overviews of changes in tissue architecture at a more gross (by major MROIs) or fine (minor MROIs) level. In brief, we manually assigned each spot in each profiled tissue section to one of 16 MROI categories (Methods), based on histology, and then automatically visualized those on rasterized vector representations of tissues for each mouse condition (Methods). In this way, we quantified spatial abundances from 100 colonic mouse sections in SPF and GF mice, spanning 10,924 spatially barcoded spots (covered by gut tissue), each with spatial expression of 17,956 host genes and 39 bacterial genera across the MROIs. On average, we sampled 20 tissue sections, 2,208 spots and ~32,000 nuclear cell segments from each mouse colon (Supplementary Fig. 10). We tested for significant spatial expression differences in the sampled sections using Splotch^36,37^ (Methods), a hierarchical probabilistic approach that accounts for the relative position of each spot (with four nearest neighbors), differences in sampling (number of spots) between MROIs and the biological batch variables of presence of bacteria in the mice (that is, conditions) and individuals (that is, animals).

### Spatial co-organization of host and microbe composition

We asked how gene expression in each of 16 MROIs was impacted by overall bacterial presence by comparing SPF versus GF mice (with no bacteria). Although both SPF and GF mice showed similar regional expression of some marker genes (for example, *Epcam* in the epithelium, *Myh11* in the muscularis regions and *Cd52* in Peyer’s patches; Fig. 3b), other genes were significantly differentially expressed between them in a region-specific manner (Fig. 3c). For example, *Satb2* and *Muc2* were, respectively, downregulated and upregulated in the crypt apex of SPF versus GF mice, the tissue layer most proximal to the mucosa and lumen (Fig. 3d). *Satb2* helps maintain intestinal homeostasis, and its expression prevents excessive crypt damage and inflammation^38^. Similarly, *Muc2* is key for maintenance of a healthy mucosal layer, and its depletion results in direct contact between epithelial cells and bacteria in the colon, leading to inflammation and cancer^39^. In other examples, *Hnf4a*, a gene associated with epithelium renewal^40^, is more highly expressed in the base of the crypt in GF versus SPF mice, and *Gpx2*, whose deficiency is related to propagating IBD symptoms^41^, is induced in the region bordering epithelium and muscularis mucosae tissue in SPF versus GF mice (Supplementary Fig. 11).

Host spatial expression patterns in SPF mice were mirrored by distinct bacterial genera detected by Splotch (Methods) at different abundances and compositions in six distinct MROIs in the SPF mice. The detected bacteria were found in the colonic inter-fold regions (crypt base, crypt mid and crypt apex/mid), the mucosal layers (crypt apex/mucosa and mucosa/pellet) or the lumen (that is, pellet, where they were most abundant, as expected). Inter-fold regions had the lowest diversity, and the pellet had the highest diversity (Fig. 3e). Morphological regions in close proximity to each other shared some highly abundant genera: *Pseudobutyrivibrio* was shared in the two mucosal regions, and *Mediterraneibacter*, an obligate anaerobe and formerly part of the *Ruminococcus* genus^42^, was shared between the inter-fold regions (Fig. 3e,f). Mucosal regions had a preponderance of *Oscillibacter* (Fig. 3f,g, middle); *Pseudobutyrivibrio* (Fig. 3f,g, bottom); and *Ruminococcus* and *Phocaeicola*, with the latter two genera previously associated with the mucosa^13,43–45^, whereas the pellet had an abundance of commensal bacteria^14,46^, such as *Lactobacillus*, *Muribaculum* and *Anaerocolumna* but also *Massilistercora*, part of the *Eubacteriales* family and previously reported only in the human gut^47^ (Fig. 3g, top). These patterns were apparent both in aggregate across samples and in individual sections, with good reproducibility (Fig. 3f–h and Supplementary Fig. 12).

**Fig. 4:**
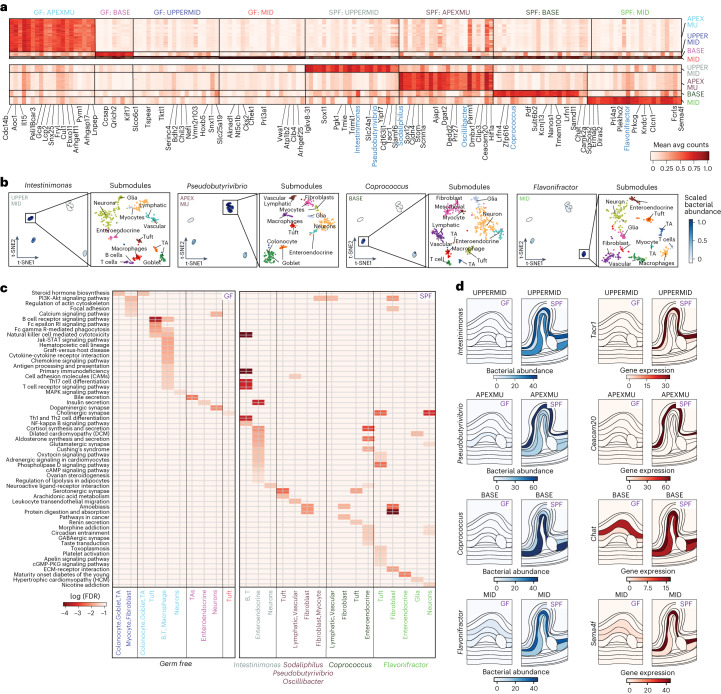
Bacterial presence influences host expression in four major tissue regions. **a**, Regional association of bacterial taxa and host gene expression. Mean average count (color scale) of selected top differentially expressed genes (columns, black text) and top differentially abundant taxa (columns, blue text) in each spatial region (rows) across four major tissue MROIs (color code, right, and labels on top). **b**, Regional association of taxa and cell type composition. Left panels: t-distributed stochastic neighbor embedding (t-SNE) of scaled bacterial count vectors in each spatial spot (dots) colored by abundance of taxa (blue color scale, scaled normalized bacterial counts, taxon on top) that are differentially abundant in each of four MROIs (color code, labels in upper left corner). Right panels: t-SNE of host snRNA-seq cell profiles (dots) mapped in each individual MROI colored by cell type label. **c**, Expression submodules in different regions reflect distinct biological processes associated with bacterial presence. Significance (color scale, −log_10_(FDR), one-tailed Fisher exact test) of enrichment of KEGG pathways (rows) in each submodule (columns) associated with each spatial region and mouse condition (color code, middle) and bacterial taxa associated with the same spatial region and condition (bottom). Color coding of spatial modules as in **a**. **d**, Differential regional gene expression associated with bacterial taxa. Region maps colored by spatial abundance of bacterial genera (blue color scale, normalized bacterial counts) and normalized spatial expression of cell type marker genes (red color scale) in each MROI in SPF and GF.

The mucosal barrier, otherwise preventing unwanted direct contact between lumen and host cells in the crypt apex, signals the immune system in a process mediated by epithelial cells^48^. We hypothesized that detected bacterial genera, some observed exclusively with tight junction mucosal barriers (for example, *Pseudobutyrivibrio*, *Ruminococcus* and *Oscillibacter*; Figs. 3f,g and 4a) and others diffusing into the tissue-specific inter-fold regions (for example, *Intestimonas*, *Coprococcus* and *Flavonifractor*; Figs. 3f and 4a), could influence and be influenced by host expression in close proximity. To systematically investigate significant regional and cell type composition differences and associate them to the presence of bacteria from different genera, we identified 28 spatial modules of genes that are co-expressed across spots (Supplementary Fig. 13a and Methods). We then partitioned each such module into gene submodules by gene co-variation across single-nucleus RNA sequencing (snRNA-seq) profiles (Fig. 4b, Supplementary Fig. 13b and Methods), recovering 203 submodules (Supplementary Table 3 and Methods). We labeled each submodule by its expression in one or multiple of the 30 cell types identified by snRNA-seq and tested it for enriched KEGG pathways (Fig. 4c and Methods).

In the presence of microbiota, specifically *Pseudobutyrivibrio*, *Sodaliphilus* and *Oscillibacter*, colonocytes in the apex of the crypts expressed *Ceacam20*, a known receptor for Gram-negative bacteria^49^ and a known colitis suppressor^50^, whereas goblet cells expressed high levels of *Hif1a*, a marker of a functioning mucosal barrier that is downregulated in IBD^51^ (Fig. 4d and Supplementary Table 4). Neurons in the neighboring region (that is, upper mid region of the crypts), in the presence of *Intestimonas*, expressed *Tacr1* and other neuroactive ligands and receptors implicated in regulating gut motility^52^, whereas macrophages in the same regions and in the presence of the same bacterial genera expressed *Fcrl2* and *Slamf6*, genes that have been shown to modulate neuro-immune signaling upon receptor–microbe binding^53,54^ (Fig. 4d and Supplementary Table 4). Specialized spatial niches in lower regions of the crypts also contained networks of neurons and myocytes involved in muscle contractility (*Camk2a* in the presence of *Coprococcus*), axon guidance (*Sema4f* in the presence of *Flavonifractor*) and cholinergic signaling (*Chat* in the presence of *Coprococcus*) (Fig. 4d and Supplementary Table 4).

## Discussion

Here we presented SHM-seq, a method that relies on solid surface capture of polyadenylated host transcripts and variable (V4) 16S bacterial regions onto spatially barcoded microarrays for joint spatial profiling of bacterial composition, host gene expression and tissue histology. We provided a deep-learning-based approach to enhance taxonomy assignment for metagenomic taxa classification from SHM-seq data with improved detection rates and assignment accuracy and a roadmap for interrogating coordinated spatial expression programs. Benchmarking against a gold standard custom reference, generated from dedicated metagenomics data in the same system, we show that SHM-seq data are compatible with mapping to different databases, containing either 16S rRNA or full genome bacterial sequences, and that the accuracy of the mapping is based on the quality and size of the respective databases.

We benchmarked the sensitivity and specificity of SHM-seq compared to traditional 16S sequencing, published RT–qPCR data as well as FISH and spatial transcriptomics in three mouse conditions: SPF, GF and ASF. SHM-seq showed reproducibility and robustness using a tissue dataset of 124 sections and detected all the bacteria genera otherwise present after 16S sequencing in SPF mice as well as all of the eight species referenced in ASF mice. Previous studies reported variation in bacterial abundance between mice^34,35,55^. In our study, we also saw differences in abundance obtained with SHM-seq versus external ASF data, although the overall correlation between the datasets was high (Pearson *r* = 0.85), and SHM-seq was highly reproducible across mice. Future studies can alter the amount and sequence of capture oligonucleotides on the spatial array surface to further tune the recovery rate of bacterial versus host transcripts or introduce other user-defined capture moieties of interest. Additionally, although sequencing only parts of the 16S rRNA gene has been shown to be sufficient to identify bacterial genera^56^, it has limited resolution at finer taxonomic levels, such as specific bacterial species and strains. SHM-seq can address these concerns in the future by modifying the capture sequences and library preparation procedures, preferably by increasing the sequencing read length.

Using these data and methods, we show that, in the presence of microbiota, subpopulations of goblet cells and colonocytes formed cell-adhesive layers filled with *Muc2* and *Ceacam20* for host–microbial communication. Additionally, we observed distinct submodules of genes expressed in specific microenvironments in SPF mice that encode proteins that can regulate intestinal physiological functions and colonic motility, which are disrupted in GF mice^57^. Thus, our spatial analysis identified spatial expression programs throughout the tissue cross-section characteristic of regional populations that display distinct, mouse-condition-relevant dynamics and may depend on the presence of commensal bacteria and/or impact host–bacteria interactions.

SHM-seq enables robust spatial host–microbiome profiling from a large number of tissues but is currently limited by the resolution of solid-phase capture arrays. To address this, Splotch, our quantitative data model (Methods), simultaneously combines spatial and experimental parameters to improve probabilistic inference of spatially resolved gene expression from lower-resolution arrays^36,37^. Moreover, by interrogating tissue contexts through MROIs, the model shares information across tissue sections to detect reproducible spatial changes in the different mouse conditions; to create a common coordinate framework (CCF) guided by the biological question and spatial resolution^58^; and to generate easier visualization of large tissue cohorts. Future studies can further tackle the resolution limitation using higher-density formats^23,24,59^ and with enhanced computational mapping approaches for deconvolving cell–cell inter-species communication networks. Additionally, using 16S rRNA databases restricted to gut microbial species can further alleviate the computational burden of mapping SHM-seq data, whereas mapping to large 16S rRNA databases increases the risk of false-positive mapping rates and the risk of lower representation of species in these databases. As such, we favor whole genome databases, such as RefSeq, and, when possible, restrict those to species present in adjacent metagenomic data, when available.

SHM-seq paves the way for future work and detailed investigation in larger studies, designed to compare animal models—for example, during colitis-induced changes^60^ or infection^61^—and human patients sampled longitudinally or cross-sectionally, where both microbiome and host cells vary, as does host genetics. Such analyses can expand understanding of the relationship between host and microbiome and lead to better understanding of mechanisms sustaining homeostasis in health or onset and persistence of chronic inflammation. Our method should, thus, help in better understanding environmental and microbiome-driven spatial neighborhood heterogeneity in barrier and mucosal tissues.

## Methods

### SHM-seq data generation

#### Mice

Adult C57BL/6 SPF mice were purchased from The Jackson Laboratory and maintained in accordance with ethical guidelines monitored by the Institutional Animal Care and Use Committee (IACUC), established by the Division of Comparative Medicine at the Broad Institute of MIT and Harvard, and consistent with the Guide for Care and Use of Laboratory Animals, National Research Council, 1996 (institutional animal welfare assurance no. A4711-01), with protocol 0122-10-16. Adult C57BL/6 GF mice were obtained from Taconic Biosciences and maintained in a gnotobiotic environment. Some of these mice were randomly selected and inoculated with ASF^33^ over several generations and used when >6 weeks of age. After colonization, ASF mice were housed in sterile conditions and tested with polymerase chain reaction (PCR) to ensure that sterility was maintained^63^. Animal housing room temperatures were monitored and always maintained according to species-specific needs. Humidity was maintained at 30–70%. Light intensity and light cycle timing were carefully regulated by Broad Institute animal facilities. To capture material from multiple sections per colonic tube, as well as to maximize the use of a single spatial array (1,007 spatial spots spread over ~42 µm^2^), we placed 2–3 tissue cross-sections onto one spatial capture area. We sampled ~20 sections from each mouse by sectioning in the aforementioned fashion across one spatial capture slide containing six active capture areas.

#### Tissue collection

Colonic tubes from the mid part of the colon were dissected within minutes of killing mice, and tissues were dried from excess fluids and embedded in Optimal Cutting Temperature (O.C.T., Fisher Healthcare) in large molds (VWR) pre-filled with O.C.T. The molds were then laid onto a metal plate pre-chilled and set on top of dry ice for 2 min or until complete freezing. Samples were transferred to −80 °C until sectioning.

#### Generation of slides with customized surfaces

Customized surface primers were immobilized to an amine-activated surface area (~40 mm^2^ each) using covalent bioconjugation^25,27^, as recommended by the manufacturer (Surmodics). Three distinct surfaces were generated for validations: 16S, poly(d)T and a mixed poly(d)T/16S surface. The oligonucleotides immobilization in each case were:

5′-[AmC6]UUUUUGACTCGTAATACGACTCACTATAGGGACACGACGCTCTTCCGATCTNNNNNNNNATCTCGACGACTACHVGGGTATCTAATCC-3′

5′-[AmC6]UUUUUGACTCGTAATACGACTCACTATAGGGACACGACGCTCTTCCGATCTNNNNNNNNTTTTTTTTTTTTTTTTTTTVN-3′ (both Integrated DNA Technologies (IDT)).

All slide incubations took place on a thermal incubator (Eppendorf Thermomixer Option C) with slides mounted into a hybridization chamber (ArrayIt). All in situ reactions performed on spatial arrays were carried out in a class II biosafety cabinet.

#### Generation of spatial arrays with customized surfaces

All spatial arrays were produced as previously described for the original spatial transcriptomics method^25,27^. In brief, six spatial microarrays per slide were created using amine-activated CodeLink slides (Surmodics). To ensure covalent binding chemistry to the amine-activated surface, DNA oligonucleotides (IDT) were constructed as follows:

5′-[AmC6]UUUUUGACTCGTAATACGACTCACTATAGGGACACGACGCTCTTCCGA TCT-[18mer spatial barcode]-[7mer random UMI]-[20T]-VN.

Printing was performed by ArrayJet LTD by spotting 100 pL of spatially barcoded DNA oligonucleotides (33 µM diluted in 2× CodeLink printing buffer) using inkjet technology to form 100-μm spots with a 200-μm spot-to-spot pitch, resulting in a total of 1,007 different spatially addressable spots printed in a 6.2-mm × 6.6-mm capture area. A complete list of all spatially barcoded DNA oligonucleotides used in this study is available at https://github.com/nygctech/shmseq. After printing the spatial arrays, slides were blocked using a pre-warmed blocking solution (50 mM ethanolamine, 0.1 M Tris, pH 9) at 50 °C for 30 min and washed with 4× saline sodium citrate (SSC) and 0.1% SDS (pre-warmed to 50 °C) for 30 min before rinsing the slides with deionized water and drying.

Next, capture areas were modified to create a customized surface containing a mixture of poly(d)T and 16S capture sequences. To hybridize the 16S probe onto the spatially barcoded poly(d)T surface probes, 75 µl of the 16S (V4) probe (IDT) with the sequence 5′-GGATTAGATACCCBDGTAGTCGAGATNBAAAAAAAAAAAAAAAAAAAA-3′ (sequence^28^ modified to enable attachment to the spatial arrays) at 0.8 nM concentration in 2× SSC (Sigma-Aldrich), 20% fresh formamide (Thermo Fisher Scientific) and 0.1% Tween (Sigma-Aldrich) was added to each spatial capture area and incubated for 30 min at room temperature. The probe mix was then removed, and capture areas were washed with 100 µl of 0.1× SSC (Sigma-Aldrich). To covalently attach the hybridized 16S probes onto the spatially barcoded poly(d)T surface probes, an extension reaction was performed with 75 µl of 1× M-MuLV buffer, 2 U µl^−1^ RNaseOUT, 20 U µl^−1^ M-MuLV and 0.5 mM dNTPs (all from Thermo Fisher Scientific) and 0.20 µg µl^−1^ BSA (New England Biolabs (NEB)) added to the wells and incubated at 42 °C for 30 min. The M-Mulv solution was then removed, followed by a wash with 100 µl of 0.1× SSC. To strip the 16S probes used in the hybridization and extension reaction, and make the covalently attached 16S surface probes single stranded, surface capture areas were incubated 3× with 75 µl of 100% formamide for 3 min at room temperature. Capture areas were then washed twice with 100 µl of 0.1× SSC before washing the entire slide for 10 min at 50 °C in 2× SSC/0.1% SDS (Sigma-Aldrich), followed by 1-min wash with 0.2× SSC and finally 0.1× SSC, both at 37 °C. This resulted in spatially barcoded capture areas containing ~1:1 ratio of poly(d)T and 16S capture sequences.

#### Cryosectioning

The entire cryo chamber, including all surfaces and tools used during cryosectioning, were wiped with 70% ethanol before the start of sectioning to avoid bacterial contamination. Both spatial arrays and O.C.T.-embedded gut tissue blocks were allowed to reach the temperature of the cryo chamber before 10-µm-thick cross-sections of gut tissue were placed on customized spatial arrays. Tissue fixation followed immediately as described below.

#### Tissue fixation, H&E staining and imaging

The spatial array was warmed at 37 °C for 2.5 min. Then, the entire area of the glass slide was covered in a methacarn solution (60% absolute methanol, 30% chloroform stabilized with ethanol and 10% glacial acetic acid (all from Sigma-Aldrich)) for 10 min at room temperature in a closed space to avoid evaporation. Methacarn was then removed, and the slide was allowed to dry before ~300 µl of isopropanol (Sigma-Aldrich) was added to the slide and incubated for 1 min at room temperature. When the slide was completely dry again, it was stained using H&E in an EasyDip Slide Jar Staining system (Weber Scientific). The system included containers separately filled with ~80 ml of Dako Mayer’s hematoxylin and Dako Blueing Buffer (both from Agilent Technologies), 5% Eosin Y in 0.45 M Tris acetate (both from Sigma-Aldrich) buffer at pH 6 and nuclease-free water (Thermo Fisher Scientific). The slide was put in a slide holder and completely dipped in hematoxylin for 6 min, followed by five dips in nuclease-free water and then 10 dips in a beaker filled with ~800 ml of nuclease-free water. The slide holder was then dipped in Dako Blueing Buffer for 5 s, followed by another five dips in nuclease-free water. Finally, the slide holder was put in the eosin solution for 1 min and washed by five dips in nuclease-free water. The slide was removed from the holder and air dried before being mounted with 85% glycerol and covered with a coverslip (VWR) before imaging. Imaging of stained H&E tissue sections on glass arrays was performed on a Metafer VSlide scanning system (MetaSystems) installed on an Axio Imager Z2 microscope (Carl Zeiss) with an LED transmitted light source and a CCD camera. Using an A-P ×10/0.25 Ph1 objective lens (Carl Zeiss) and a configuration program^26^, focusing and scanning of each tissue section on the glass array was done automatically. Image stitching was done using VSlide (version 1.0.0) with 60-µm overlap and linear blending between fields of view. Images were extracted using jpg compression.

#### In situ reactions: permeabilization and reverse transcription

Before start, the hybridization chamber was cleaned with RNaseZap (Thermo Fisher Scientific) and 70% ethanol, followed by at least 30 min in a UV light chamber. After section imaging, the slide was again attached to the hybridization chamber to proceed with the following permeabilization reactions (referred to as ‘bacterial treatment’ below). First, 100 µl of a lysozyme solution with 0.05 M EDTA (pH 8.0, Thermo Fisher Scientific), 0.1 M Tris HCl, pH 8 (Thermo Fisher Scientific) and 10 µg µl^−1^ lysozyme (from chicken egg white, lyophilized powder, Sigma-Aldrich) were added to each well and incubated for 30 min at 37 °C, followed by wash with 100 µl of 0.1× SSC. Second, 75 µl of 10% Triton X-100 (Sigma-Aldrich) was added and incubated for 5 min at 37 °C, followed by a 100-µl wash of 0.1× SSC. Third, a solution with 0.05% SDS and 5 mM DTT (Thermo Fisher Scientific) was added and incubated for 5 min at 37 °C, followed by a 100-µl wash of 0.1× SSC. Fourth, 100 µl of collagenase I (200 U) in 1× HBSS (both from Thermo Fisher Scientific) were added to each well and incubated for 20 min at 37 °C, again followed by a 100-µl wash of 0.1× SSC. Lastly, 75 µl per well of 0.1% pepsin (pH 1, Sigma-Aldrich) was incubated for 10 min at 37 °C, followed by a final wash of 100 µl of 0.1× SSC. In situ cDNA synthesis was performed as previously described^26^. In brief, 75 µl of 50 ng µl^−1^ actinomycin D (Sigma-Aldrich) and 0.5 mM dNTPs (Thermo Fisher Scientific, 0.20 µg µl^−1^ BSA and 1 U µl^−1^ USER enzyme (both from NEB), 6% v/v Lymphoprep (STEMCELL Technologies), 1 M betaine (B0300-1VL, Sigma-Aldrich), 1× first-strand buffer, 5 mM DTT, 2 U µl^−1^ RNaseOUT and 20 U µl^−1^ Superscript III (all from Thermo Fisher Scientific)) were added to each well. The reaction was sealed with Microseal ‘B’ PCR Plate Seals (Bio-Rad) and incubated for at least 6 h. After incubation, 70 µl of the released cDNA material from each hybridization chamber well was collected and stored in a 96-well PCR plate (Eppendorf).

#### Library preparation

Library preparation was performed using the SM-Omics automated library preparation protocol, as previously described^26^. In brief, released cDNA material was first made double stranded using the nicked RNA template strands as primers for copying the cDNA strand with DNA polymerase I. To avoid overdigestion, the reaction was terminated with EDTA, and ends were blunted using T4 DNA polymerase before linear amplification by in vitro transcription. Amplified material was again transcribed into cDNA, resulting in material ready for PCR indexing as described in the next subsection.

#### Quantification, indexing and sequencing

qPCR quantification and indexing were performed as previously described^64^ using TruSeq LT Illumina indexing and a KAPA HotStart HiFi ReadyMix (Roche). Indexed cDNA libraries were cleaned using a 0.7:1 ratio with AMPure XP beads (Beckman Coulter) to PCR product, according to the manufacturer’s protocol, and eluted in 12 µl of elution buffer (Qiagen). Each sample’s concentration was measured using the DNA HS Qubit assay (Thermo Fisher Scientific), and average fragment length was determined using either Bioanalyzer HS or DNA1000 TapeStation (both from Agilent Technologies). Each sample was then diluted to the desired concentration for sequencing (1.08 pM on a NextSeq and 10 pM on a MiSeq, both with ~10% PhiX). Pooled libraries were sequenced with 25 nucleotides (nt) in the forward read and 55 nt and 150 nt in the reverse read on NextSeq and MiSeq (Illumina), respectively.

### Generation of bacterial validation data

#### Mechanical extraction of bacterial RNA

An approximately 1-mm-thick tissue section with pellet was sectioned from SPF colons in O.C.T. and put in a dry ice-cold Lysis Matrix D tube (MP Biomedicals). Then, 400 µl of RLT buffer (Qiagen) with 1% 2-mercaptoethanol (Sigma-Aldrich) was added to the tube, and the solution was homogenized in a FastPrep-24 instrument (MP Biomedicals) at speed 6 for 40 s. Tubes were then centrifuged for 5 min at 12,000 r.p.m. Supernatant was transferred to a new tube, and RNA extraction was done using the RNeasy Mini Kit (Qiagen), according to the manufacturer’s instructions. Extracted RNA was fragmented using the NEBNext Magnesium RNA Fragmentation Module Kit (NEB), heating for 2 min. Fragmented RNA was cleaned with the MinElute Cleanup Kit (Qiagen), according to the manufacturer’s instructions. Quality of the fragmented RNA was evaluated by the Bioanalyzer Pico Kit (Agilent Technologies). Next, ~20 ng µl^−1^ mechanical extracted RNA was added on a 16S surface probe coated quality control (QC) array in an in situ cDNA reaction, as described in the ‘In situ reactions: permeabilization and reverse transcription’ subsection. After at least 6-h incubation at 42 °C, 70 µl of the released material from each well was collected and stored in a new 96-well PCR plate (Eppendorf). Library preparation, quantification, indexing and sequencing on the MiSeq were performed as described in the ‘Library preparation’ and ‘Quantification, indexing and sequencing’ subsections.

#### Extraction and metagenomic sequencing of fecal DNA

Pellet was collected from the colon of SPF mice by perforating the colon wall and scraping the pellet and mucus into a 1.5-ml collection tube (Eppendorf). Collected pellet was stored at −80 °C until further processed. DNA was extracted from the pellet using a Lysing Matrix Y tube (MP Biomedicals), according to the manufacturer’s instructions. Extracted DNA concentration was determined using the DNA HS Qubit assay. DNA was made into libraries using Nextera XT (15031942 v05). Concentration and average fragment length of each sample were evaluated using the DNA HS Qubit assay (Thermo Fisher Scientific) and Bioanalyzer HS (Agilent Technologies), respectively. Each sample was diluted to the desired concentration for sequencing (9 pM, ~10% PhiX), and pooled samples were sequenced on a MiSeq (2 × 150 bp, lllumina). Each sample was sequenced to ~5–10 million reads.

#### FISH

FISH was performed on the same fresh-frozen gut tissue samples from ASF mice. All sections were 10-µm-thick cross-sections and consecutively collected. First sections were placed on the spatial array, followed by placing consecutive sections on a CodeLink amine-activated slide (Surmodics); the following two sections were then again placed on the spatial array. Sections on the spatial array were used for SHM-seq, and sections on the amine-activated CodeLink slide (Surmodics) were prepared for FISH as further described. Slides were warmed at 37 °C for 2.5 min on a thermal incubator, before tissue sections were fixed using freshly prepared methacarn, as described in the ‘Tissue fixation, H&E staining and imaging’ subsection. Slides were then placed in a hybridization chamber, and 75 µl of preheated FISH solution (0.9 M NaCl and 20 mM Tris, pH 7 (both Thermo Fisher Scientific), 0.1% SDS (Sigma-Aldrich) and a FISH oligonucleotide detection probe (0.06 µg ul^−1^)) was added to each well and incubated for 2 h at 25 °C. Oligonucleotide detection FISH probes (IDT) were used depending on the target of interest: probe EUB338 (5′-/Cy5/GCTGCCTCCCGTAGGAGT-3′) for all bacteria; probe non-338 (5′-/Cy5/ACTCCTACGGGAGGCAGC-3′) as a negative control; probe Lab158 (5′-/Cy5/GGTATTAGCAYCTGTTTCCA-3′)^65–67^ to target ASF360; probe Lac435 (5′-/Cy5/TCTTCCCTGCTGATAGA-3′)^68,69^ to target ASF502; and probe Bac303 (5′-/Cy5/CCAATGTGGGGGACCTT-3′)^8,67^ to target ASF519. After the 2-h incubation, FISH solution was removed, and wells were washed with 100 µl of 1× PBS before the hybridization chamber was removed and slides were dipped 12 times in 50 ml of 1× PBS before being air dried. Slides were mounted with 85% glycerol (Sigma-Aldrich) and a coverslip (VWR). Epifluorescent images were acquired on an Axio Imager Z2 microscope using a PhotoFluor LM-75 light source (89North) in combination with a Plan-APOCHROMAT ×63/1.4 oil DIC objective (Carl Zeiss). Images were processed using VSlide (version 1.0.0, MetaSystems).

### Processing on H&E imaging data

#### Image registration and annotation

Image processing and registration of barcoded spots was done using SpoTteR^26^. H&E images (collected in RGB channels) were downscaled to approximately 500 × 500 pixels. For efficient grid spot detection, tissues were masked from the images using quantile thresholding in the red channel. Centroids of spatial array spots were detected by computing the image Hessian. Centroid coordinates were used as probable grid points, and a rectangular grid was then fitted to these probable points using a local optimizer (nlminb, R package stats (R version 3.6.3)). With iterations and removing 10% of the probable spots that did not fit the perfect grid structure, a new grid was fitted until the target number of grid points per row (here, 35) and column (here, 33) was reached. Final grid points were overlapped with the previously masked tissue section to select spatial points present only under the detected tissue section area. These points were used in further analysis.

H&E images were annotated using a graphical cloud-based interface^24^ by manually assigning each spatial coordinate (*x*,*y*) resulting from the grid fitting process with one or more morphological region tags. The tags used were epithelium (E), epithelium and muscle and submucosa (ALL), epithelium and mucosae and submucosa (EMMSUB), epithelium and mucosae (EMM), muscle and submucosa (MSUB), crypt base (BASE), externa and interna (MEI), externa (ME), interna (MI), mucosae and interna (MMI), mucosa and pellet (MUPE), crypt mid (MID), crypt apex and mucosa (APEXMU), crypt apex and crypt mid (UPPERMID), Peyer’s patch (PP) and pellet (PE). E, EMMSUB, EMM, BASE, MEI, ME, MI, MUPE, MID, MMI, APEXMU, UPPERMID, PP and PE were visualized in tissue vector representations.

### Processing of host reads

#### Raw reads processing and mapping of host reads

Reads were generated with bcl2fastq2 (version 2.20.0) and trimmed to remove adaptor sequences and the 16S surface probe sequence using BBDuk^70^ (version 38.33). ST Pipeline (version 1.7.6)^29^ was used to generate gene-by-barcode matrices. The reverse quality-filtered reads were mapped with STAR (version 2.6.0)^71^ to the mouse genome reference (GRCm38 primary assembly), and mitochondrial sequences were removed. Mapped reads were annotated using HTseq-count (version 0.11.4)^72^ and the mm11 mouse annotation reference (https://www.gencodegenes.org/mouse/release_M11.html). Annotated reads were demultiplexed with TagGD^29,73^ (version 0.3.6) with a Hamming distance clustering approach (k-mer 6, mismatches 2). This connected transcript information to spatial barcodes. Finally, UMI collapsing per transcript and spatial barcode was performed with a naive clustering approach (mismatches 1) similar to that described in UMI-tools^74^.

### Processing of bacterial reads

#### Generation of gold standard mouse gut bacterial reference

FASTQ reads were generated with bcl2fastq2, and reads were quality filtered using KneadData (version 0.7.4) (https://huttenhower.sph.harvard.edu/kneaddata/) (mouse database mouse_C57BL). MEGAHIT^75^ (version 1.2.9) was used for assembly of the filtered reads, and bowtie2 (ref.^76^) (version 2.3.4.3) was used for mapping reads to the assembly. MetaBAT2 (ref.^77^) (version 2.15) was used for binning the assembly, and the command-line version of NCBI BLAST^78^ (version 2.9.0+) was used to assign taxonomy to contigs with blastn and database ‘nt’. MEGAHIT, bowtie2 and MetaBAT2 were all run using default settings. Assignments were filtered (E-value ≤ 10E−6) and sorted (by E-value and percent identity), and each contig was then assigned the top taxonomy assignment. Contigs belonging to an assigned taxonomy on species level at various cutoffs (>0.1%, >0.05% and >0.01% corresponding to 65, 121 and 419 species, respectively) were retained. For each cutoff, reference genomic sequences (complete genomes, chromosomes or scaffolds, depending on availability for these species) were downloaded from the NCBI RefSeq database^31^ (release 205), resulting in FASTA sequence databases (one for each cutoff) of the taxa found in SPF mice (*n* = 6) and used as input to build custom databases in Kraken2 (version 2.0.9)^30^ according to Kraken2 default instructions, including masking of low-complexity regions. Reference genomes for six species were not found in the RefSeq database (Supplementary Table 5) and were not included in the FASTA sequence databases. The mouse gut bacterial references were also filtered for genera that have previously been found in mice and/or the intestine^79–81^. A phylogenetic tree of the reference taxa was built using NCBI’s Common Tree and visualized using iTOL (version 6.4.3)^82^. When analyzing mouse gut tissue with defined flora (ASF), genome sequences according to ref. ^83^ were downloaded from the NCBI and used as input to build a custom ASF database in Kraken2.

#### Generation of simulated data

Two simulated datasets were generated based on the abundance of taxa using cutoffs 0.1% and 0.01% (as described in the ‘Generation of gold standard mouse gut bacterial reference’ subsection): 16S rRNA FASTA sequences for the taxa found in SPF mice were downloaded from the NCBI (downloaded 24 July 2021), except two taxa where the 16S rRNA FASTA sequence were missing (*Sodaliphilus pleomorphus* and *Anaerocolumna sedimenticola*). Command-line NCBI BLAST^78^ (version 2.9.0+) was used to align every possible sequence version of the 16S surface probe to the 16S rRNA FASTA sequences to find the best possible alignment for the 16S surface probe per taxa. To mimic spatially captured reads from a real SHM-seq, 2 million paired reads from a real SHM-seq experiment were used as a template for FASTQ headers, sequence and quality scores for the forward read and FASTQ headers and quality scores for the reverse read. The sequences in Read 2 were replaced by 150-bp-long fragments of the 16S rRNA sequences from randomly selected taxa. Fragments were created by selecting a region upstream of the best possible alignment of the 16S surface probe per randomly selected taxa. Each region was then randomly selected a length based on a normal length distribution with parameters characteristic to a spatial array (400 ± 44 bp) and trimmed to 150 bp. This resulted in a simulated dataset with 2 million randomly selected 16S rRNA gene sequences, generated from where the 16S surface probe was expected to capture, from the taxa in our mouse gut bacterial references but with known exact taxa and both reverse and forward reads.

#### Deep learning model: data pre-processing

A total of 500,000 DNA sequences were randomly selected from the simulated dataset based on a 0.1% abundance cutoff (described in the ‘Generation of simulated data’ subsection) and uniformly sampled, and single-point mutations with 0.1% rate were introduced. This was followed by random shortening based on a normal distribution of fragment lengths from a true SHM-seq experiment (143 ±13 bp, truncated at 150 bp). Reads from each taxon in the mouse gut bacterial reference were represented at least 100 times per genus. Sequences were one-hot encoded, such that each nucleotide (A, C, T, G and N) was represented by a five-dimensional binary vector, followed by sequence padding up to the maximum length (150 bp). Taxa labels were one-hot encoded into one of N genera. The encoded sequences and taxa labels were provided as input for training the model.

#### Deep learning model: architecture

A taxonomic classifier of short reads was implemented using Keras^84^ with TensorFlow^85^ back end (version 2.2.0) in Python (version 3.8.10) (Supplementary Fig. 4a). The model takes as input one-hot encoded DNA sequences of varying lengths and provides a genus label as output. First, a masking layer was used to ignore padded entries, followed by four layers of a one-dimensional convolutional layer with kernel sizes of 15, 17, 19 and 23 to extract short motifs, followed by a concatenation and a dropout (50% rate) module and two bidirectional long short-term memory network layers, which processed the sequences in both directions. This was followed by another dropout layer (20% rate), a dense layer (reLU activation), a dropout layer (10% rate), another dense layer (reLU activation) and, finally, a fully connected layer (softmax activation) to reduce the final output size to the number of distinct genera in the input data. In total, the model consisted of 298,760 trainable parameters. Cross-entropy loss was used to train a multi-class classifier with Adam as the optimization algorithm^86^. The model architecture was visualized using Netron^87^.

#### Deep learning model: training details

Model parameters were optimized by using 80% of sequences for training and 20% for testing. Each epoch started with shuffling the training data and computing the gradient update once for each training data point to obtain unbiased gradient estimates^88^. During training, categorical accuracy and cross-entropy loss were used to monitor progress. Training was terminated after a maximum of 15 epochs or when the training loss did not decrease in five consecutive epochs. The area under the receiver operating characteristic (ROC) curve and the F1 score were calculated using Scikit-learn (version 0.24.2)^89^ and used to report the final performance on test data.

#### Deep learning model: evaluation

One million simulated sequences with corresponding taxa (as in the ‘Generation of simulated data’ subsection) were modified with a sequencing error rate of 1%^90^ and random shortening as described above. Sequences were classified either by Kraken2 alone or by Kraken2 followed by the deep learning model. Performance was evaluated compared to the ground truth taxa labels by calculating Bray–Curtis dissimilarities and Pearson correlation coefficients of the bacterial relative abundances per spot using Scipy (version 1.1.0)^91^ spatial.distance.braycurtis and Scikit-learn (version 0.24.2)^89^ stats.pearsonr, respectively. A higher similarity of the relative abundances between classifications and the ground truth resulted in lower Bray–Curtis dissimilarities and higher Pearson correlations. Accuracy and F1 score were calculated on the whole dataset using Scikit-learn (version 0.24.2)^89^ metrics.classification_report.

#### Comparison of taxonomy assignments

To compare how well Kraken2 performs when using different RefSeq databases (whole genome versus 16S rRNA) of different sizes (restricted versus unrestricted), taxonomy assignments were made by the taxonomy assignment pipeline but without using the deep learning model (as described in the ‘Raw reads processing and mapping of bacterial data’ subsection). The four databases used in the comparisons were: RefSeq Bacteria whole genome database (downloaded from Kraken2 GitHub version 2.1.2) and adding to it the whole genomes from all eight ASF species in Kraken2 (ref.^83^) (‘RefSeq whole genomes’); the custom gold standard restricted whole genome database (‘65 species whole genome’, as described in the ‘Generation of gold standard mouse gut bacterial reference’ subsection) and the RefSeq Bacteria 16S rRNA database, derived from those RefSeq bacterial taxa that had available 16S rRNA sequences in the NCBI (‘RefSeq 16S rRNA’, ~3,000 taxa, downloaded on 24 July 2021); and finally, we restricted the RefSeq 16S rRNA database to the 65 species detected in the gold standard restricted whole genome database (‘65 species 16S rRNA’). For comparing the impact of read lengths, simulated datasets were prepared as described in the ‘Generation of simulated data’ subsection by using cutoff 0.1% but with longer length distribution (650 ± 44 bp) and trimmed to 150 bp, 300 bp, 450 bp and 600 bp.

#### Raw reads processing and mapping of bacterial data

FASTQ reads were generated with bcl2fastq2 and trimmed to remove adaptor sequences using BBDuk^70^. Trimmed reads were quality filtered using the same quality-filtering step as in the ST Pipeline (version 1.7.6)^29^, but only reads longer than 100 nt were kept. TagGD^73^ was used to connect the spatial barcode to each forward read (k-mer 6, mismatches 2, Hamming distance clustering algorithm), and BWA-MEM (version 0.7.17)^92^ with reference mouse genome (GRCm39) was used to remove host mapping sequences. Remaining reverse reads were mapped to the mouse gut bacterial reference (created as described in the ‘Generation of gold standard mouse gut bacterial reference’ subsection) using Kraken2 (version 2.0.9)^30^ (confidence 0.01). Reads originated from GF and SPF mice were mapped to the mouse gut bacterial reference, whereas reads originated from ASF mice were mapped to the ASF reference. Taxonomy assignments made by Kraken2 were improved using the deep learning model. UMIs with identical spatial barcodes and taxonomical assignments were collapsed using UMI-tools (version 1.0.0)^74^ (UMIClusterer, threshold 1), resulting in a bacteria-by-barcode matrix.

### Analysis of bacterial validation data

#### Spatial analysis of bacterial fluorescence

Bacterial presence in scanned fluorescence images was detected using ilastik (version 1.3.3)^93^. After training and testing each bacterial fluorescence print separately in ilastik, the resulting bacterial detection mask was aligned with the fluorescent image to detect mean fluorescence intensity per spatial coordinate and stored as a matrix. This matrix was then run in Splotch (as described in the ‘Hierarchical probabilistic modeling using Splotch’ subsection). Resulting normalized fluorescence intensity was compared to the normalized bacterial presence by randomly selecting, at most, three spatial coordinates from each annotated region per sample (only annotated regions that were shared between the normalized fluorescence intensity and the normalized bacterial presence were considered) and scaling them within each sample, before matching them to a spatial coordinate in the same region and comparing them to each other (normalized fluorescence intensity versus normalized bacterial presence per spatial coordinate). To limit the region annotated as pellet, spatial coordinates annotated as pellet were selected if they were spatially adjacent to coordinates annotated as mouse tissue. This procedure was repeated 1,000 times to generate an average spatial correlation measurement between normalized bacterial FISH intensity and normalized sequenced bacterial presence, expressed as Spearman correlation.

#### 16S surface probe sensitivity

To evaluate 16S surface probe sensitivity, reference DNA sequence and gene annotation files were downloaded from Ensembl Bacteria^94^ for the ASF bacteria available in the database (version 104.1) (ASF356, ASF360, ASF457, ASF492, ASF500 and ASF519 (taxonomy ID 1235789)). Reads captured from ASF tissue sections on a spatial transcriptomics QC array with only 16S surface probes on the array surface were separately mapped against each ASF bacteria genome using BWA-MEM (version 0.7.17)^92^. Gene body coverage over the 16S rRNA genes in respective reference genomes was generated using RSeQC (version 4.0.0)^95^. Genome binning was done by summarizing the aligned reads in separate bins, each bin representing a hundredth of the respective ASF genome.

#### 16S surface probe specificity

Specificity was first evaluated by proportion of bacteria versus mouse read alignment. Tissue sections from SPF, ASF and GF mice were placed on QC arrays with 16S surface probes, and finished libraries were prepared using either bacterial treatment or colon treatment. Each finished library was sequenced to approximately 660,000 reads. Reads were taxonomically annotated by using the taxonomy assignment pipeline without the deep learning model. The proportion of reads mapping to the respective bacterial reference (mouse gut bacterial reference for SPF and GF tissue samples and ASF reference for ASF tissue samples) was calculated by using the number of trimmed reads.

Protocol specificity was also evaluated by comparing the bacterial treatment with a mechanical treatment (see the ‘Mechanical extraction of bacterial RNA’ subsection). Spearman rank and Pearson correlation coefficients were calculated using Scipy’s (version 1.1.0)^91^ stats.spearmanr and stats.pearsonr.

Bacterial treatment was compared to a bulk 16S rRNA sequencing dataset^62^ where the 16S libraries were made from material originating from feces of C57BL/6J mice (Sequence Read Archive (SRA) sample references: SRR9212951, SRR9213178 and SRR9213335). The correlation was calculated using Scipy’s (version 1.1.0) Pearson correlation coefficient^91^.

#### Comparison of the taxonomy assignment pipeline with QIIME 2

The taxonomy assignment pipeline (as described in the ‘Raw reads processing and mapping of bacterial data’ subsection) was compared to QIIME 2 (ref.^32^) (version 2022.2) by using the simulated dataset (generated as described in the ‘Generation of simulated data’ subsection). QIIME 2 was run with default settings for single-end sequences, and the Silva 138 99% OTUs full-length sequences classifier was used for taxonomic profiling.

#### Effect of bacterial treatment on mouse gene expression

To evaluate the effect of the bacterial treatment on measured host (mouse) gene expression, we normalized^96^ gene counts from samples with and without bacterial treatment (reads downsampled to the same saturation levels) and from samples prepared on a spatial array with customized surface or a standard spatial array (reads downsampled to the same saturation levels). Pearson correlation coefficient was calculated using Scipy’s (version 1.1.0)^91^ stats.pearsonr.

### Spatial modeling and visualization of host–microbiome data

#### Hierarchical probabilistic modeling using Splotch

Splotch^36,37^ was used for statistical analysis of spatial data. Splotch is a hierarchical probabilistic that captures variation in spatial transcriptomics data through modeling of different study design covariates, such as individual’s age or mouse condition (${\rm{B}}$); a linear model component capturing spatial variation in array data with a conditional autoregressive (CAR) prior (${{\psi }}$); and gene expression variation captured in each independent spatial measurement (${{\epsilon }}$) to account for technical artifacts. Sequencing depth is accounted for by using a size factor *s* where the total number of captured UMI counts per spatial spot is divided by the median UMI counts across all analyzed spots. The posterior distribution of the parameters is interrogated from the model—for example, when the model was conditioned of bacterial presence in the tissues to quantitate expression changes across both the mouse conditions and different tissue contexts.

Genes (*i*), tissue sections (*j*) and independent spatial spots (*k*) were indexed as follows: $$i\in \left[\mathrm{1,2},\ldots ,{N}_{\text{genes}}\right],j\in \left[\mathrm{1,2},\ldots ,{N}_{\text{tissues}}\right],$$$$k\in \left[\mathrm{1,2},\ldots ,{N}_{\text{spots}}^{\left(\;j\right)}\right].$$ Gene expression in each spot is considered an approximation of observed counts $${y}_{i,\,j,k}$$, where $${y}_{i,\,j,k}$$ is expected to equal to $${s}_{j,k}{{{\lambda }}}_{i,\,j,k}$$. $${s}_{j,k}$$ is the size factor (total number of UMIs observed at spot *k* and tissue section $$j$$, and $${\lambda }_{i,\,j,k}$$ is the rate of gene expression (referred to as normalized counts throughout)). Splotch then models the observed counts using the zero-inflated Poisson (ZIP) distribution:

1 $${y}_{i,\,j,k} \sim {\mathrm{ZIP}}\left({s}_{j,k}{\lambda }_{i,\,j,k},{\theta }_{i}^{\,p}\right),{\mathrm{nb}}=0,{\mathrm{zi}}=1,$$

where ${{{\theta }}}_{i}^{p}$ represents the gene-specific probability of a dropout. The zero-inflated models account for an overabundance of zeros by introducing a second zero-generating process gated by a Bernoulli random variable:

2 $$\begin{array}{l}{y}_{i,\,j,k}\sim\left\{\begin{array}{l}\begin{array}{ll}\qquad0,\quad\qquad\qquad{if}\,{\theta }_{i}=1,\end{array}\\ \begin{array}{l}{\mathrm{Pois}}\left({s}_{j,k}{\lambda }_{i,\,j,k}\right),\quad{if}\,{\theta }_{i}=0\end{array}\end{array}\right.\\ \qquad\qquad\quad{\theta }_{i}^{\,P}\sim{\mathrm{Beta}}\left(1,2\right),\\ \qquad\qquad\quad{\theta }_{i}\sim{\mathrm{Bernoulli}}\left({\theta }_{i}^{\,P}\right),\end{array}$$

where the Poisson process can be replaced by negative binomial (NB) without loss of generality. The gene expression rate parameter *λ*_*i,j,k*_ is described in terms of a generalized linear model (GLM) by three components:

3 $$\log \left({{{\lambda }}}_{i,\,j,k}\right)={B}_{i,\,j,k}+{{{\psi }}}_{i,\,j,k}+{{{\epsilon }}}_{i,\,j,k}.$$

where ${B}_{i,\,j,k}$ is the characteristic expression of gene *k* within the context of spot *k*, from which a characteristic expression vector ${{{\beta }}}_{i}\in {R}^{{N}_{\text{MROI}}}$ is derived describing which MROI spot *k* comes from. At the top level, the dataset is split along an important covariate (for example, presence of bacteria), and a separate ${{{\beta }}}_{i,{l}_{1}}$ is modeled for each unique group (${l}_{1}\in \{1,\ldots {L}_{1}\}$). At the next level, each set is further partitioned along another covariate (for example, animal individual). A two-level hierarchical model for ${{{\beta }}}_{i}$ can, thus, be specified as:

4 $$\begin{array}{l}{{{\beta }}}_{i,{l}_{1}} \, \sim \, {\mathscr{N}}\left(0,{\left({{{\sigma }}}_{i}^{\left({l}_{1}\right)}\right)}^{2}I\right),\\ {{{\beta }}}_{i,{l}_{1},{l}_{2}} \, \sim \, {\mathscr{N}}\left({{{\beta }}}_{i,{l}_{1}},{\left({{{\sigma }}}_{i}^{\left({l}_{2}\right)}\right)}^{2}I\right),\\ {{{\sigma }}}_{i}^{\left({l}_{2}\right)} \, \sim \, {{\mathscr{N}}}_{\ge 0}\left(0,1\right),\end{array}$$

where, in practice, ${{{\sigma }}}_{i}^{\left({l}_{1}\right)}=2$ for all $i,{l}_{1}$, and posteriors are inferred over all ${{{\sigma }}}_{i}^{\left({l}_{2}\right)}$. For convenience, because each tissue $j$ belongs to one covariate group at each level, the inverse mapping function ${{{\rho }}}^{-1}\left(\,j\right)$ is introduced that maps $j$ to the appropriate *l*_1_,*l*_2_,*l*_3_ indices for *β*_*i*_. With this in hand, *B*_*i,j,k*_is formally defined in the non-compositional model:

5 $${B}_{i,j,k}={x}_{j,k}^{T}{{\rm{\beta }}}_{i,{{\rm{\rho }}}^{-}1\left(j\right)},$$

where ${x}_{j,k}$ is a one-hot encoding of the spot MROI annotation ${D}_{k}^{\left(\;j\,\right)}$ used to index the relevant entry in the characteristic expression vector ${{{\beta }}}_{i,{{{\rho }}}^{-}1\left(\;j\,\right)}$.

*ψ*_*i,j,k*_ describes the how the local and immediate neighborhood of spot *k* has an effect gene *i* and is modeled using the CAR prior. The observations in each spatial spot are assumed to be dependent on the spot’s immediate spatial neighborhood defined as four nearest neighbors. *ψ*_*i,j,k*_ is defined as a Markov random field over the spots in each array:

6 $$\begin{array}{ccc}{{{\psi }}}_{{\rm{i}},{\rm{j}}}{\rm{|}}{{{\alpha }}}_{{\rm{i}},{\rm{j}}},{{{\tau }}}_{{\rm{i}},{\rm{j}}},{{\rm{W}}}_{{\rm{j}}}\sim{\mathscr{N}}\left(0,{\left({\tau }_{i}{K}_{j}\left(I-{\alpha }_{i}{K}_{j}^{-1}{W}_{j}\right)\right)}^{-1}\right),\\ {{{\alpha }}}_{i}\sim{\mathscr{U}}\left(0,1\right),\\ {{{\tau }}}_{i}\sim {\Gamma }^{-1}\left(1,1\right),\end{array}$$

where ${{{\sigma }}}_{i}$ is a spatial autocorrelation parameter; ${{{\tau }}}_{i}$ is a conditional precision parameter; ${K}_{j}$ is a diagonal matrix containing the number of neighbors for each spot in tissue $j$; and ${W}_{j}$ is the adjacency matrix (with zero diagonal).

${{{\epsilon }}}_{i,j,k}$ captures variation at the level of individual spots with the assumption that each spot was independently and identically distributed (i.i.d) to infer their standard deviations:

7 $$\begin{array}{l}{{{\epsilon }}}_{i,\,j,k} \, \sim \, {\mathscr{N}}\left(0,{{{\sigma }}}_{i}^{2}\right),\\ {{\sigma }}{\rm{\_}}i \, \sim \, {N}_{\ge 0}(0,0.{3}^{2}),\end{array}$$

where *σ*_*i*_ is the inferred level of variability for gene $j$.

Data were processed as a two-level model when describing differences in mouse model/condition and morphological region (when comparing SPF versus GF mice) or as a one-level model for ASF mouse analysis. Input data were raw UMI counts (as described above). Sampling from the posterior was done running four independent chains with 200 iterations per chain (100 warmup and 100 sampling). The model was conditioned on 10,924 spots, 16 morphological region tags and two mouse conditions (SPF versus GF) (two-level model) or 4,397 spots, five morphological region tags and one mouse condition (ASF) (one-level model).

For differential expression analysis, each pairwise comparison of gene expression was denoted as a random variable Δ_β_ that describes the difference between two conditions as *β*_1_ − *β*_2_. *β*_1_ and *β*_2_ represent any two conditions arising from any two combinations in the model—for example, any two genes, sample covariates (for example, mouse condition; SPF versus GF) or MROIs regions (for example, crypt apex and mucosa versus crypt base). The null hypothesis presumes that the two posterior distributions over characteristic expression coefficients *β*_1_ and *β*_2_ estimated by the model are identical and that Δ_β_ is tightly centered around zero. To quantify this similarity, Δ_β_|${\mathcal{D}}$ (where ${\mathcal{D}}$ is the training data) is compared to the prior distribution Δ_β_ using the Savage–Dickey density ratio^97^ that estimates the Bayes factor (BF) between the conditions:

8 $$\text{BF}\approx \frac{p\left({\Delta }_{\beta }=0\right)}{p\left({\Delta }_{\beta }=0|{\mathcal{D}}\right)},$$

where the probability density functions are evaluated at zero. If expression is different between the two conditions, then the posterior Δ_β_|${\mathcal{D}}$ will not be centered around zero, and the estimated BF will be large; hence, the null hypothesis is rejected, and the two genes are denoted as differentially expressed between the conditions. Hereafter, the Savage–Dickey density ratio is referred to as BF. Upregulated genes (Δ_β_ > 0) with at least log(BF) > 0.5 were considered as differentially expressed between any two conditions and used in all downstream analysis. Bacterial genera were called as detected in SPF tissue if the bacterial weighted mean count per morphological region was greater than the maximal weighted mean in corresponding morphological mouse region in GF. The total regional count had to count for more than 2% of the total bacterial count to be called as detected.

#### Visualizing expression and abundances with rasters

To enable spatial data visualization across sections and conditions, a rasterized tissue representation of canonical tissue architecture of the mid part of the colonic tube was created as scalable vector graphics (svg) and annotated with MROI information. Tissue vectors captured the two most common tissue architectures observed in this study (a zoomed-out view of major MROIs (E, EMM, ME, MEI, MI, MMI, PP, MUPE and P) and a zoomed-in view of minor MROIs (APEXMU, BASE, MID, UPPERMID, EMM, EMMSUB, ME, MEI, MI, MMI, PP, MUPE and P)) and used only for visualizations. matplotlib^98^ was used to automatically plot averaged host gene or bacterial expression from all spatial spots corresponding to each MROI and condition as annotated in the svg files.

### Host gene expression mapped using cell type signatures

#### snRNA-seq data processing

Mouse colon snRNA-seq data were obtained from ref. ^99^, containing 340,461 individual cell profiles across 22,986 expressed genes. In brief, nucleus profiles with >800 genes expressed in a minimum of 10 cells and <30% mitochondrial or rRNA signatures were retained for analysis. Raw counts data were normalized to transcripts-per-10,000 (TP10K). To regress out genes as differentially expressed, the mean and the coefficient of variation (CV) of expression of each gene were calculated and partitioned into 20 equal-frequency bins. LOESS regression was used to estimate the relationship between log(CV) and log(mean), and genes with the 1,500 highest residuals were equally sampled across these bins. To account for differences in batches, this was performed for each sample separately, and a consensus list of 1,500 genes with greatest recovery rates was selected. Next, using Scanpy^100^, Harmony^101^ was used for further batch correction with 20 neighbors and 40 principal components from principal component analysis. After 10 iterations, convergence was reached, and the resulting data were clustered with PhenoGraph^102^, with 25 nearest neighbors using the Minkowski metric. Cell type labels provided in ref. ^99^ were used to manually label clusters after PhenoGraph clustering.

#### Spatial co-expression analysis and definition of modules

All posterior estimates that account for both morphological differences and differences in mouse conditions were used as *λ*_*i,j,k*_ in a sparse matrix format $\varLambda \in {R}^{{N}_{{\mathrm{spots}}}{{xN}}_{{\mathrm{genes}}}}$, where *N*_spots_ = 5,413 and *N*_genes_ = 17,956. The snRNA-seq normalized counts and SHM-seq posterior means counts tables were standardized separately across cells and spots within genes, respectively, considering common genes (*N*_common genes_ = 16,525) in both datasets, resulting in matrices ${X}_{{\mathrm{standardized}}}\in {R}^{{N}_{{\mathrm{cells}}}{xN}{}_{{\mathrm{common}}\;{\mathrm{genes}}}}$ and ${\varLambda }_{{\mathrm{standardized}}}\in {R}^{{N}_{{\mathrm{spots}}}{{xN}}_{{\mathrm{common}}\; {\mathrm{genes}}}}$. Finally, the similarity of each cell to each spot $P$ was calculated as the Pearson correlation coefficient *r* between its standardized and imputed expression vector (columns of ${X}_{{\mathrm{standardized}}}$) and spots’ expression vectors (columns of ${\varLambda }_{{\mathrm{standardized}}}$), resulting in cell-specific similarity vectors. Morphological spots were used from all region categories except for those found in PE and MUPE. To find sets of co-expressed genes—that is, with similar spatial patterns across spots—the data *P* were hierarchically clustered with the average linkage method using the L1 norm (Manhattan distance), with a set distance threshold to detect 28 distinct blocks (subsets of genes co-expressed across subsets of spots—hereafter, spatial modules) using scipy.cluster.hierarchy.fcluster.

#### Using snRNA-seq profiles to partition modules to submodules

Gene expression submatrices were created of the expression of genes belonging to each spatial co-expression module. To identify which specific cell types underlie expression in each spatial module or submodule, mean expression values were calculated for each gene across the single-cell profiles in each of 30 snRNA-seq clusters (as described in the ‘snRNA-seq data processing’ subsection) and scaled by dividing each gene’s mean expression per cluster by its maximum mean expression across cell type clusters. Genes with an average scaled expression lower than 1 were removed (scaled expressions set to 0). Then, to estimate cell type compositions in each spatial module (or submodule), the expression profiles in each spatial module for the subset of genes from the 30 filtered and averaged snRNA-seq cell type (cluster) signatures were hierarchically subclustered within each spatial module using cosine distance and average linkage. These genes of each module were then grouped in submodules using 0.4× the maximum of the linkage matrix as cutoff. Next, two-sided Wilcoxon signed-rank test (followed by Benjamini–Hochberg false discovery rate (FDR)) was used to compare enrichment of cell type (cluster) signatures in the co-expression submodules in a one-versus-rest fashion. The cell types used in the enrichment analysis were: neurons, transit amplifying cells (TAs), cycling TAs, myocytes, goblet cells, colonocytes, fibroblasts, glia, lymphatic cells, macrophages, enteroendocrine cells, mesothelial cells, stem cells, T cells, tuft cells, B cells and vascular cells.

#### KEGG pathway enrichment

KEGG database^103^ gene sets were tested for enrichment in each cell-type-specific submodule with a one-tailed Fisher exact test followed by a Benjamini–Hochberg FDR. KEGG pathways with FDR < 0.05 were visualized.

### Reporting summary

Further information on research design is available in the Nature Portfolio Reporting Summary linked to this article.

## Online content

Any methods, additional references, Nature Portfolio reporting summaries, source data, extended data, supplementary information, acknowledgements, peer review information; details of author contributions and competing interests; and statements of data and code availability are available at 10.1038/s41587-023-01988-1.

## Supplementary information

Supplementary Information Supplementary Figs. 1–13 and Supplementary Information References.

Reporting Summary

Supplementary Table 1 Spatial metadata.

Supplementary Table 2 Host and bacterial raw counts metric per spatial spot.

Supplementary Table 3 Cell-type-specific submodule expression.

Supplementary Table 4 KEGG pathway analysis for all modules and submodules. One-tailed Fisher exact test values are reported.

Supplementary Table 5 Species excluded from gold standard reference.

## Data Availability

All raw data have been deposited to NCBI’s SRA under accession PRJNA999495 (ref.^104^). All processed data have been deposited in the Single Cell Portal under accession SCP2375 (https://singlecell.broadinstitute.org/single_cell/study/SCP2375).

## Code availability

All code is deposited on GitHub at https://github.com/nygctech/shmseq (ref.^105^).
