# Supplementary material for: Spatial host–microbiome sequencing reveals niches in the mouse gut
Source: Nat Biotechnol. 2023 Nov 20;42(9):1394–403. doi: 10.1038/s41587-023-01988-1 (PMC11392810; doi:10.1038/s41587-023-01988-1)
Supplement: Supplementary file 1 — Supplementary Figs. 1–13 and Supplementary Information References. [file 41587_2023_1988_MOESM1_ESM.pdf]

nature biotechnology

Article https://doi.org/10.1038/s41587-023-01988-1

Spatial host–microbiome sequencing reveals niches in the mouse gut

In the format provided by the authors and unedited

Supplementary Figure 1. Array saturation. 2
Supplementary Figure 2. Spatial architecture of tissue sections on a SHM-seq array. 3
Supplementary Figure 3. Comparison of mouse gut bacterial references. 4
Supplementary Figure 4. Deep learning (DL) model performance. 6
Supplementary Figure 5. Reads captured by the 16S surface probe align to the expected 16S rRNA gene locations in selected bacteria in the ASF mouse model. 8
Supplementary Figure 6. 16S surface probe captured the expected part of the 16S rRNA gene in selected bacteria in the ASF mouse model. 9
Supplementary Figure 7. Bacterial abundances estimated by SHM-seq in ASF mice are consistent across replicates and with qPCR. 10
Supplementary Figure 8. SHM-seq validation by fluorescence in situ hybridization of select bacterial targets in ASF colon sections. 11
Supplementary Figure 9. Spatial gene expression performance metrics are comparable between SHM-seq and traditional Spatial Transcriptomics (ST) in SPF and ASF mouse tissue sections. 12
Supplementary Figure 10. Sampling metrics for 100 colonic mouse sections (SPF and GF mice). 13
Supplementary Figure 11. Differential spatial gene expression across mouse models and MROIs. 14
Supplementary Figure 13. Spatial expression modules. 16
Supplementary Information References 17

Supplementary Figures

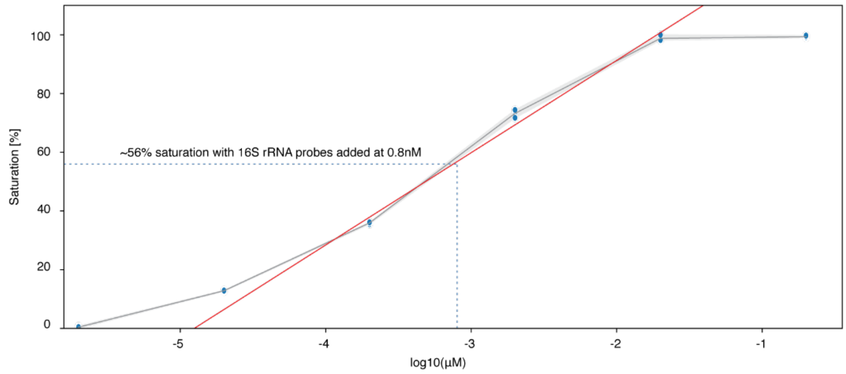

Supplementary Figure 1. Array saturation. Dilution curve showing surface saturation with the 16S primer based on scaled qPCR generated Cq values (y axis, 0-100% saturation) and the $\log_{10}$ molarity (x axis, 16S dilution series) (n=3, blue dots). Red line: linear regression of the slope in the linear area of the dilution curve. Blue dashed lines: $\log_{10}$(molarity) of the 16S probe (x axis) and its corresponding saturation level (y axis).

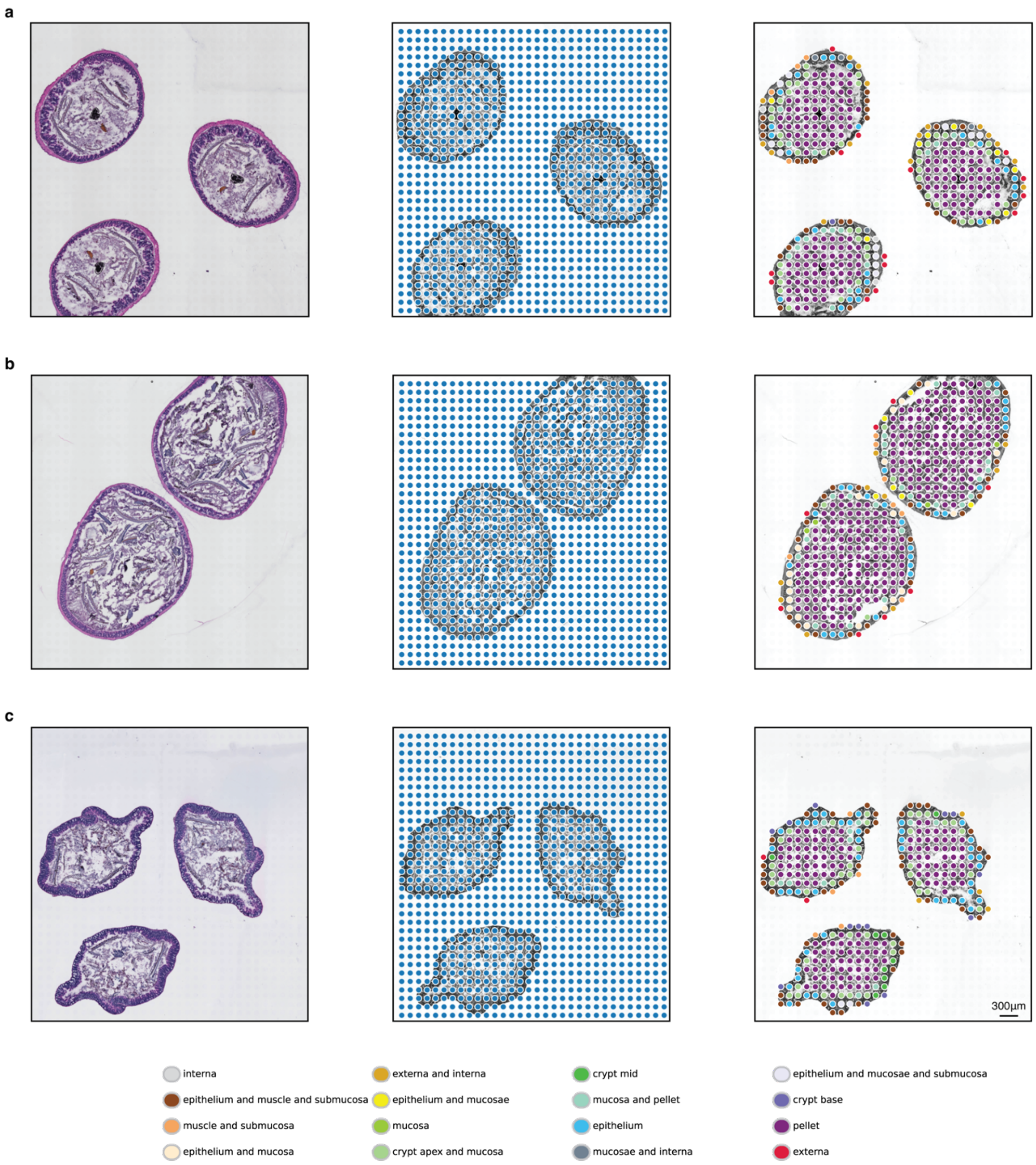

Supplementary Figure 2. Spatial architecture of tissue sections on a SHM-seq array. Example cross sections of colon tissue from mice stained by hematoxylin and eosin (H&E) (left) and overlaid with 1,007 spatial spots in the spatial capture area (middle) or with morphological annotations (color code) (right) for SPF (**a**), ASF (**b**) or GF (**c**) mice. Experiments were repeated 52 times. (**a-c**) Scale bar: 300µm.

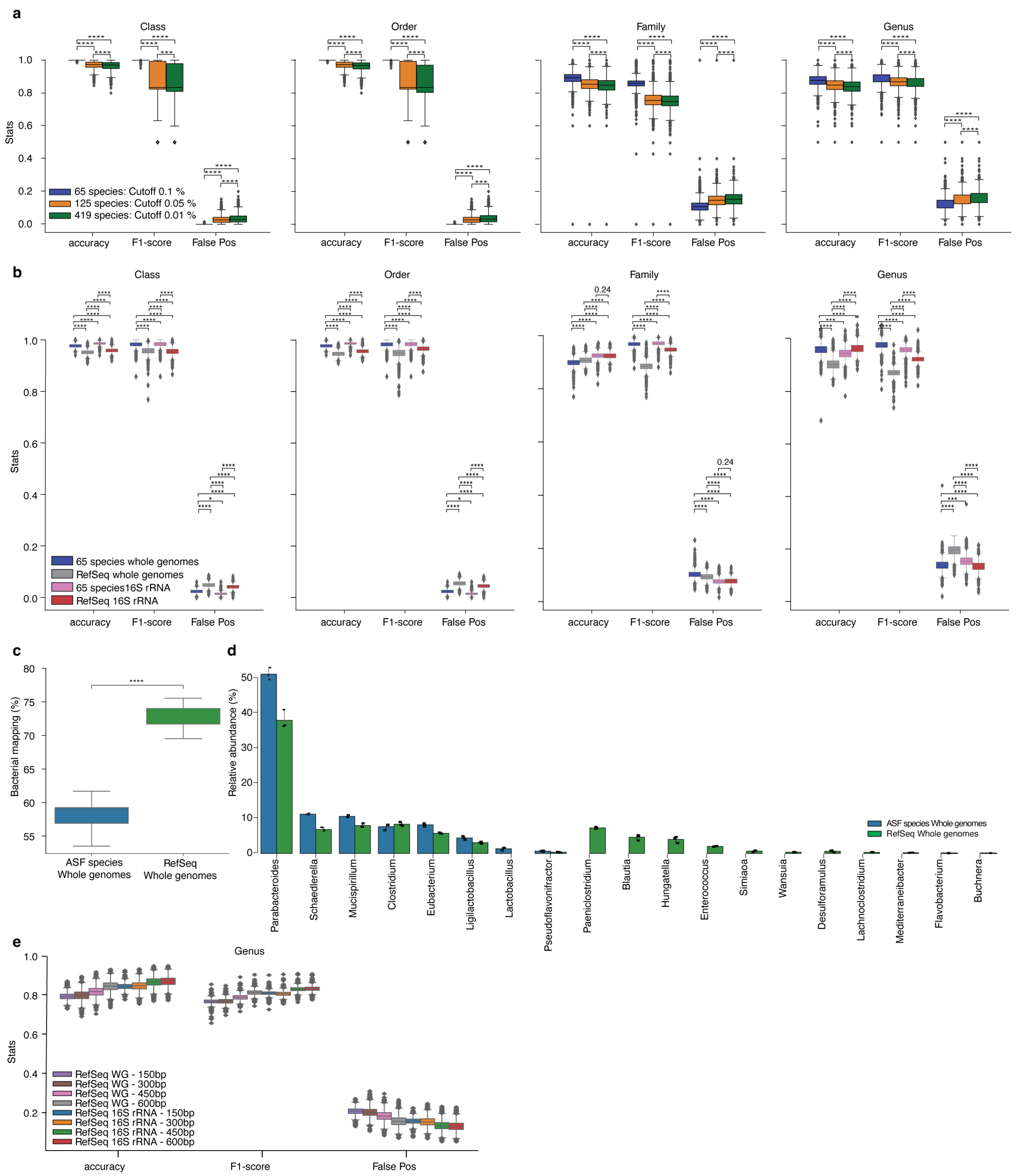

Supplementary Figure 3. Comparison of mouse gut bacterial references. (a) Impact of number of species in the Gold standard reference on accuracy and sensitivity. Distribution of values (y axis) for accuracy, F1 score and false positive rate (x axis) when using Kraken2 on a simulated dataset with reads from 419 (green; >0.01% abundance), 125 (orange; >0.05%) or 65 (blue, >0.1%) species detected in metagenomic sequencing on four taxonomic levels (panels, labels on top) (n = 3, technical replicates). **(b)** Impact of reference on accuracy and sensitivity. Distribution of values (y axis) for accuracy, F1 score and false positive rate (x axis) when using Kraken2 at each of four taxonomic levels (panels; labels on top) using a simulated dataset with reads from 65 species from all spatial spots (x axis) using a Gold standard reference (blue, as **(a)**), RefSeq whole genome database (~20,823 bacterial genomes, gray), RefSeq 16S database for the 65 species (65 species 16S rRNA, pink) or RefSeq 16S database of ~3,000 taxa that had 16S sequences in NCBI (RefSeq 16S rRNA, red) (n = 3, technical replicates). **(c,d)** Impact of reference on bacterial mapping rate and species abundance estimates. Bacterial

mapping rate (**c**, y axis) and relative abundance of specific species (**d**, y axis) in real ASF data (n = 3, biological replicates, black dots) when using Kraken2 and either an ASF whole genome bacteria reference[1] (blue) or RefSeq whole genomes (~20,823 whole bacterial and 8 ASF species, green). Error bars in (**d**) represent 95% confidence intervals. (**e**) Impact of read length and reference on accuracy and sensitivity. Distribution of values (y axis) for accuracy, F1 score and false positive rate (x axis) for simulated sequencing data at different read lengths (150, 300, 450 or 600bp) classified at the genus level by Kraken2 using either the RefSeq whole genome (~20,823 genomes) or RefSeq 16S databases (~3,000 taxa) (as in **b**) (n = 3, technical replicates). Box plots (**a, b, c, e**): Center black line, median; color-coded box, interquartile range; error bars, 1.5x interquartile range; black dots; outliers. (*) $10^{-2}<p\leq0.05$, (***) $10^{-4}<p\leq10^{-3}$, (****) $p\leq10^{-4}$, two-sided *t*-test.

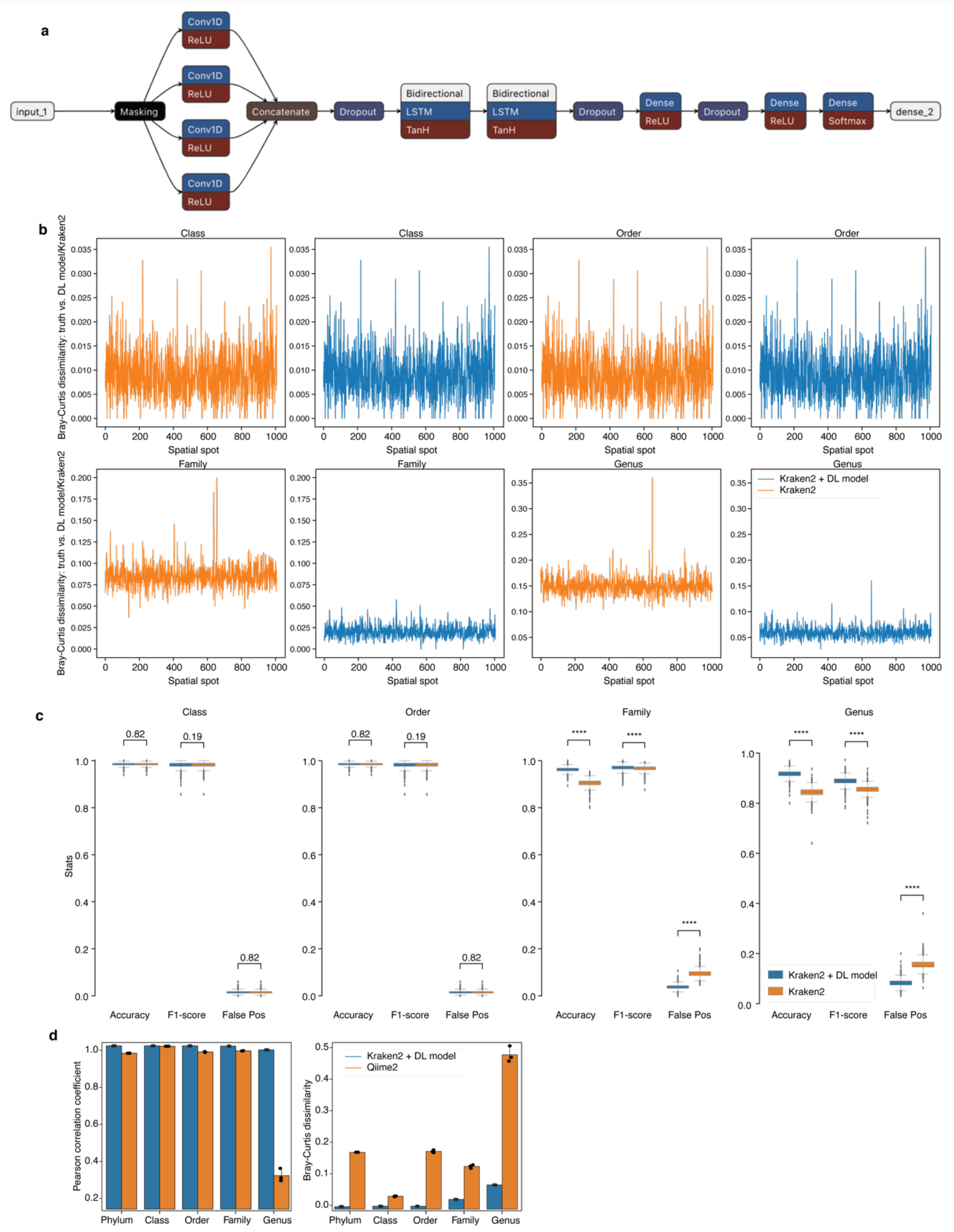

Supplementary Figure 4. Deep learning (DL) model performance. (a) Overview of DL model architecture. The model uses five-dimensional binary vectors to represent the nucleotides and these are first masked to ignore padded entries, followed by four layers of a one-dimensional convolutional layer with increasing kernel-sizes. After the convolutional layers, a concatenation and a dropout followed. The model then used two bidirectional Long Short Term Memory networks to process the sequences in both directions, before another dropout layer. This was followed by first a dense layer (reLU activation), second a dropout layer, third another dense layer (reLU activation) and finally a dense layer (softmax activation).

(**b-d**) Performance comparison between Kraken2 and Kraken2+DL model. **(b)** Bray-Curtis dissimilarities (y axis) between true data labels and taxonomic classification in each spatial spot (x axis) at four taxonomic levels (panels) using Kraken2 (orange) or Kraken2 + DL model (blue). **(c)** Distribution of scores (y axis) for accuracy, F1 and false positive rate (x axis) for simulated sequencing data classified at four taxonomic levels using either Kraken2 (orange) or Kraken2 + DL model (blue) (n = 3, technical replicates). (****) $p \leq 10^{-4}$, two-sided t-test. Center black line, median; color-coded box, interquartile range; error bars, 1.5x interquartile range; black dots; outliers. (**d**) Average Pearson's *r* (*y* axis, left) and average Bray-Curtis dissimilarities (*y* axis, right) between true and predicted taxonomic labels from all spatial spots on five taxonomic levels (x axis) from our taxonomy assignment pipeline (Kraken2 + DL) using the mouse gut bacteria reference (blue) or from Qiime2 (orange) using a 16S rRNA reference (n = 3, black dots). Error bars: 95% confidence intervals.

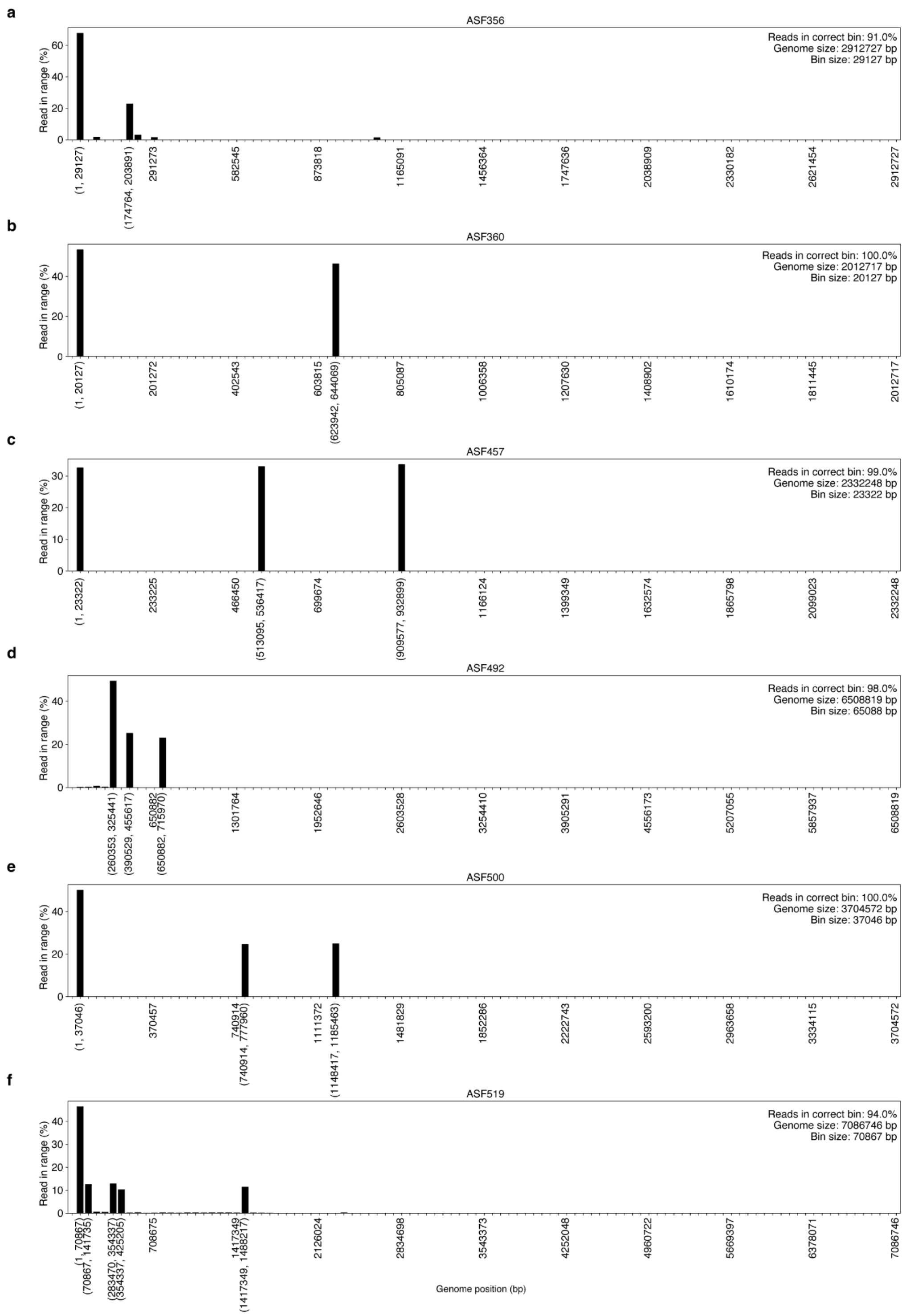

Supplementary Figure 5. Reads captured by the 16S surface probe align to the expected 16S rRNA gene locations in selected bacteria in the ASF mouse model. Proportion of reads (y axis) at each genome location interval with >10% of detected reads (x axis) in the ASF365 **(a)**, ASF360 **(b)**, ASF457 **(c)**, ASF492 **(d)**, ASF500 **(e)** and ASF519 **(f)** genome.

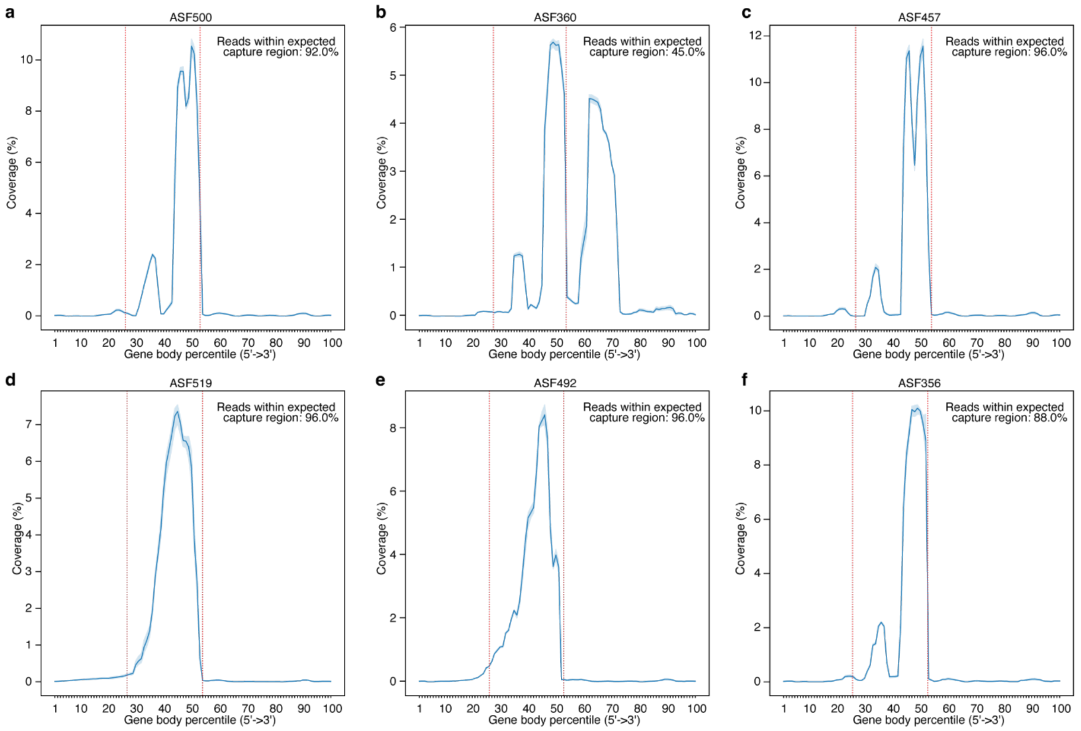

Supplementary Figure 6. 16S surface probe captured the expected part of the 16S rRNA gene in selected bacteria in the ASF mouse model. Mean gene body coverage (y axis, n = 3) at each position (x axis) of 16S rRNA genes shown in **(a)** ASF500, **(b)** ASF360, **(c)** ASF457, **(d)** ASF519, **(e)** ASF492 and **(f)** ASF356. Red vertical lines: expected captured region of the 16S rRNA gene. Shaded area: 95% confidence interval.

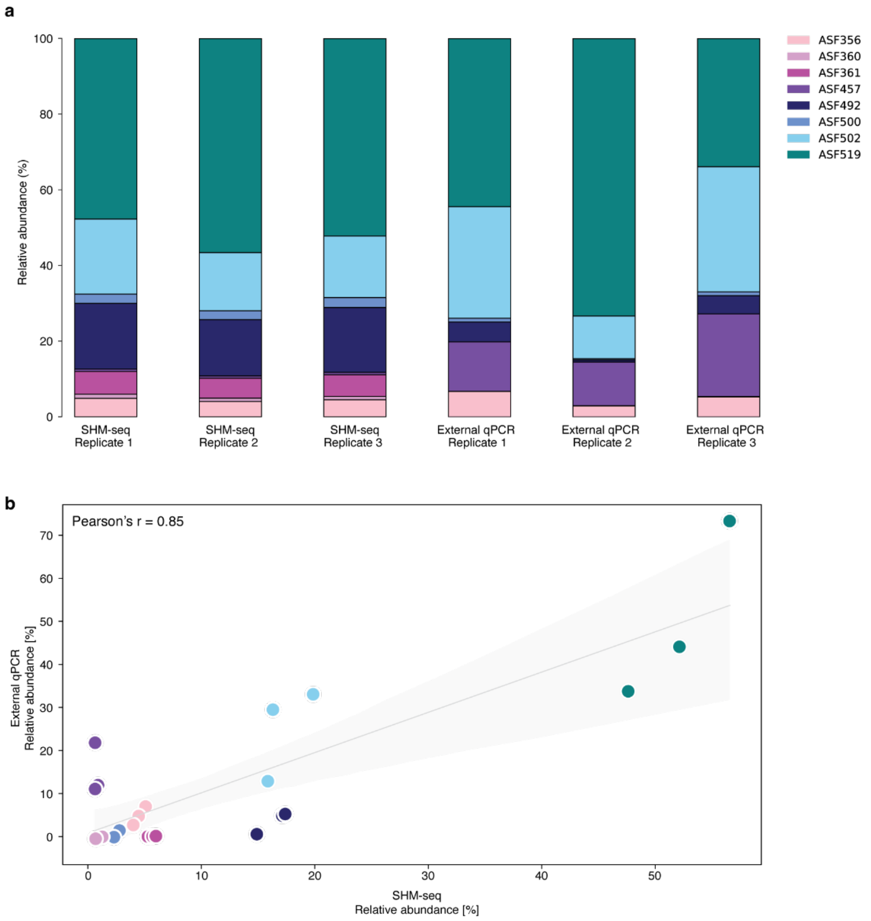

Supplementary Figure 7. Bacterial abundances estimated by SHM-seq in ASF mice are consistent across replicates and with qPCR. (a) Relative abundance (y axis) estimated for each ASF species from ASF mice measured by bacterial reads in SHM-seq (n = 3) or RT-qPCR count data[2] (external reference). **(b)** Bacterial read abundances for each ASF species (color code as in **a)** from SHM-seq of ASF tissue sections (n = 3, x axis) or RT-qPCR count data[2] (y axis). Line: linear regression model fit. Shaded area: 95% confidence interval.

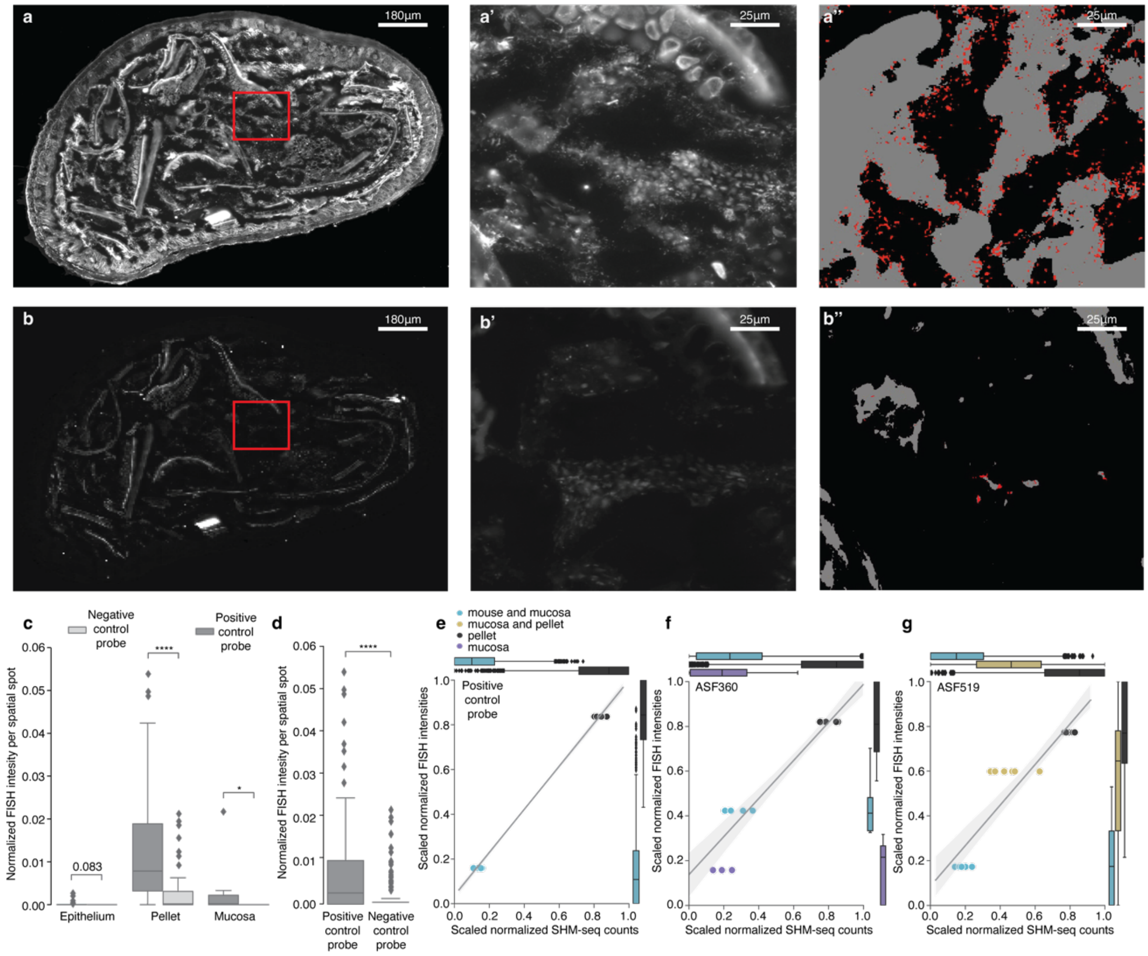

Supplementary Figure 8. SHM-seq validation by fluorescence in situ hybridization of select bacterial targets in ASF colon sections. (a-b) Raw fluorescent signal from a FISH experiment using a positive (**a**) or negative (**b**) control probe in a cross section of an ASF mouse colon (**a, b**) and zoomed-in region (**a’,b’**) along with (**a’’,b’’**) bacterial locations (red) and fibers (gray) as classified using Ilastik (**Methods**). **(c)** Distribution of normalized bacterial fluorescence intensity per spatial spot (y axis) in three MROIs (x axis) (Epithelium, positive probe n = 38, negative probe n = 39; Pellet, positive probe n = 50, negative probe n = 67; Mucosa, positive probe n = 15, negative probe n = 5) for positive (dark gray) and negative (light gray) control probes. **(d)** Distribution of normalized bacterial fluorescence intensities per spatial spot (y axis) for positive (n = 105, left) and negative (n = 131, right) control probes. **(e-g)** Mean scaled normalized fluorescence intensity from FISH (y axis) and scaled normalized bacterial count generated using SHM-seq (x axis) in each MROIs and sample (dots, color code in **e**), n=6, biological replicates) **(Methods)** using **(e)** positive control FISH probe, **(f)** FISH probe targeting ASF360 and **(g)** FISH probe targeting ASF519. Color coded boxplots on top and right: Distribution of normalized signals per MROI category. (**a-b**) Scale bar: 180µm. (**a’-b’’**) Scale bar: 25µm. **(c-g)** Boxplots: Center black line, median; color-coded box, interquartile range; error bars, 1.5x interquartile range; black dots; outliers. **(e, f, g)** Shaded areas: 95% confidence interval. Line: linear regression model fit. (*) $0.01<p\leq0.05$, (****) $p\leq10^{-4}$, two-sided t-test. Experiments were repeated 5 times.

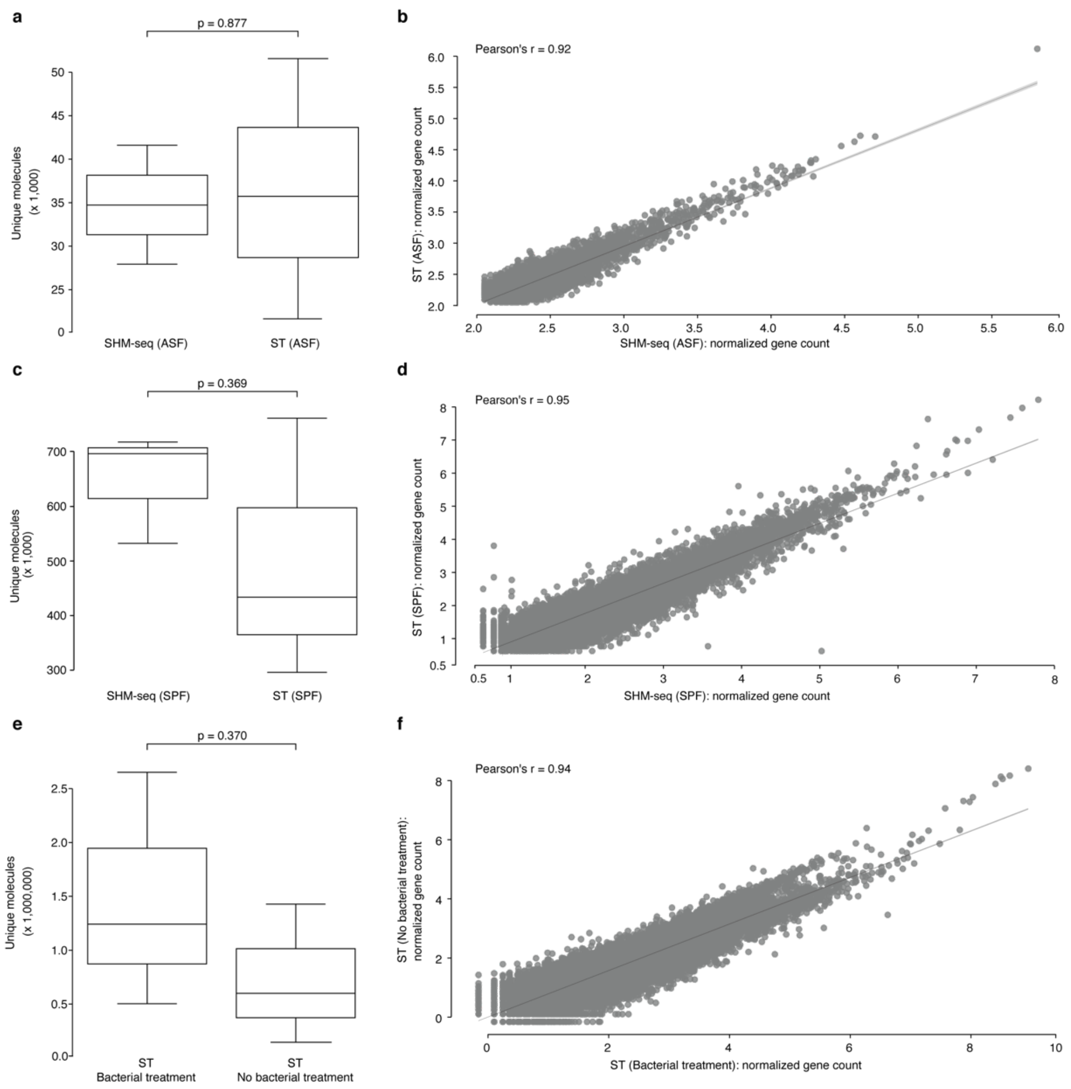

Supplementary Figure 9. Spatial gene expression performance metrics are comparable between SHM-seq and traditional Spatial Transcriptomics (ST) in SPF and ASF mouse tissue sections. (a) Distribution of the total number of UMIs (y axis) from ASF mice using SHM-seq (left, n = 3) and ST (right, n = 3). **(b)** Normalized gene expression counts in the ASF mouse model using SHM-seq (x axis, n = 3) and ST (y axis, n = 3). **(c)** Distribution of the total number of UMIs obtained (y axis) from SPF mice using SHM-seq (left, n = 3) and ST (right, n = 3). **(d)** Normalized gene expression counts in the SPF mouse model using SHM-seq (x axis, n = 3) and ST (y axis, n = 3). **(e)** Total number of UMIs in the SPF mouse model in ST with (left, n = 3) and without (right, n = 3) applying bacterial treatment *in situ* **(Methods)**. **(f)** Normalized gene expression in SPF mice profiled by ST with (x axis, n = 3) and without (y axis, n = 3) applying bacterial treatment *in situ* **(Methods)**. p values: two-sided t-test. Boxplots: Center black line, median; box, interquartile range; error bars, 1.5x interquartile range; black dots; outliers.

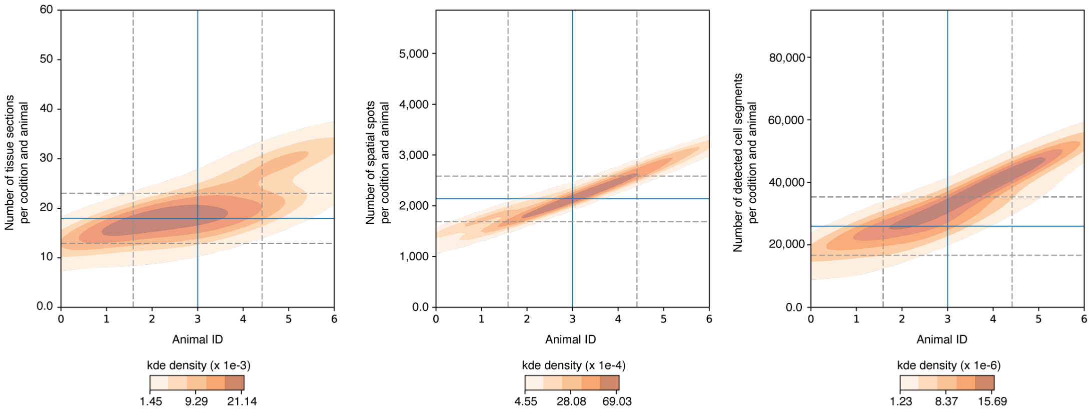

Supplementary Figure 10. Sampling metrics for 100 colonic mouse sections (SPF and GF mice). Number of tissues (y axis, left), spatial spots (y axis, middle), and cell segments (y axis, right) sampled in each of the mice (x axis) in the SPF (n=3) and GF (n=3) models. Blue lines; median number of observations per condition, gray dashed lines; standard deviations for all observations per condition.

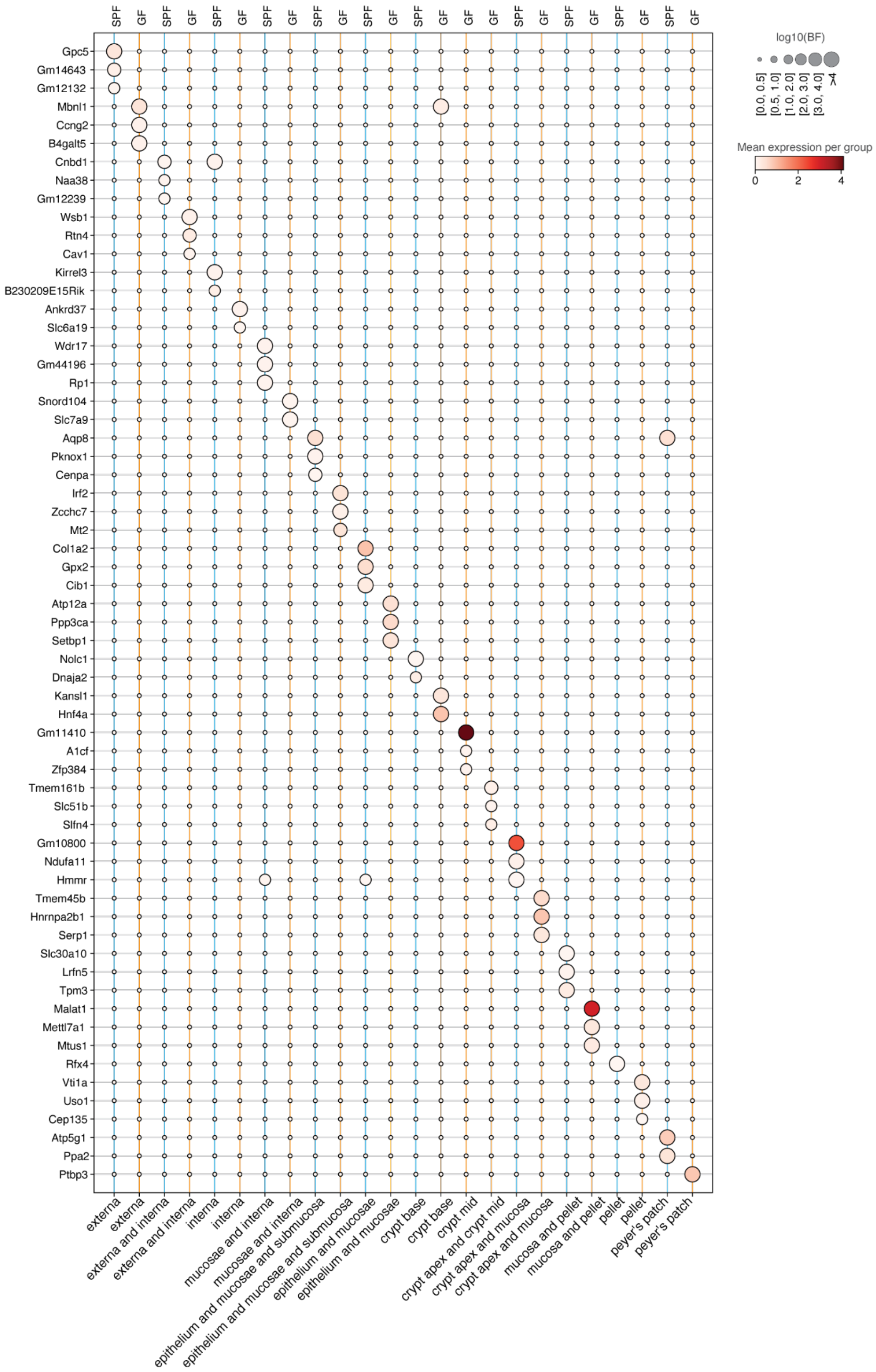

Supplementary Figure 11. Differential spatial gene expression across mouse models and MROIs. Significance (dot size; $\log_{10}$(Bayes Factor)) and effect size (dot color: normalized expression) for each of the top 3 genes (rows) differentially expressed between conditions (GF *vs.* SPF) for each MROI (columns).

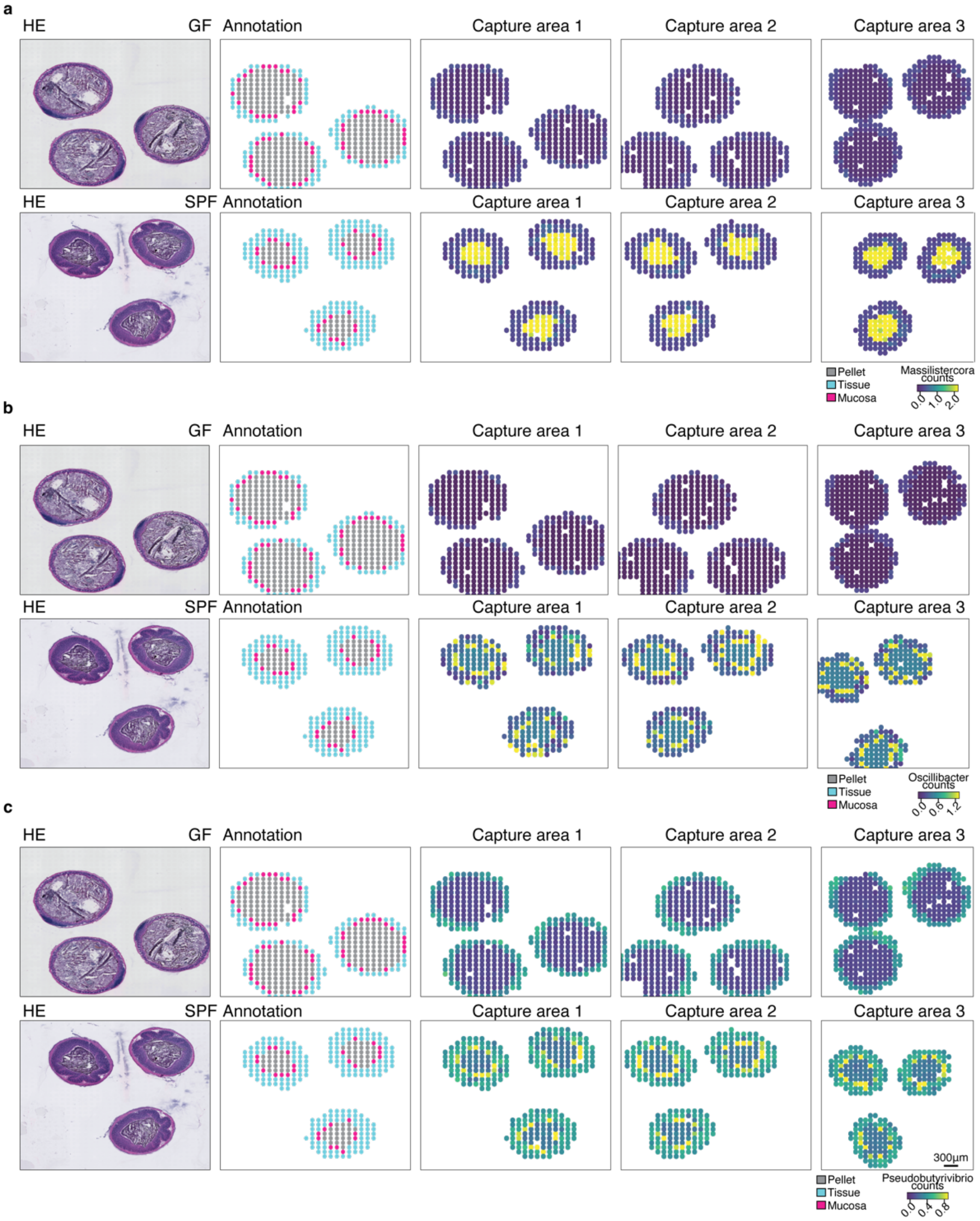

Supplementary Figure 12. Reproducibility of bacterial abundances across spatial spots, tissue sections and capture areas. From left: H&E image (from capture area 1, leftmost), MROI annotations (second left, color code), and normalized bacterial counts in three capture areas (middle, second right and rightmost, color bar) for individual sections from GF (top) and SPF (bottom) mice, for *Massilistercora* (**a**), *Oscillibacter* (**b**) and *Pseudobutyrivibrio* (**c**) genera. Experiments were repeated 36 times for SPF and 54 times for GF conditions, respectively. (**a-c**) Scale bar: 300µm.

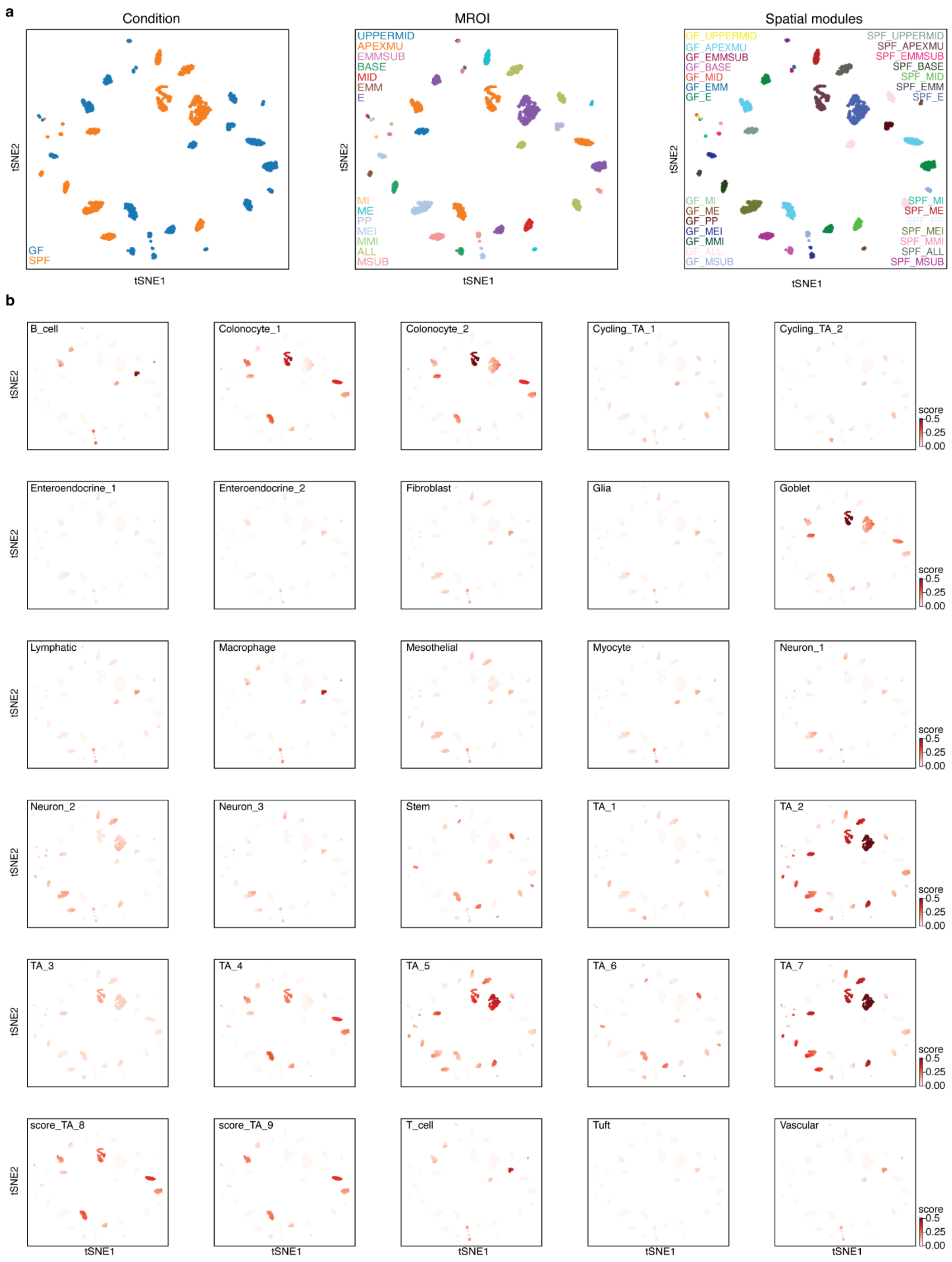

Supplementary Figure 13. Spatial expression modules. t-distributed stochastic neighbor embedding (t-SNE) of SHM-seq spot profiles colored by mouse condition (a, left), MROI (a, middle), spatial module assignment (a, right), or score of expression signature of different cell types (b, color scale; cell type name on top left of each panel).

Supplementary Information References

1. Wannemuehler, M. J., Overstreet, A.-M., Ward, D. V. & Phillips, G. J. Draft genome sequences of the altered schaedler flora, a defined bacterial community from gnotobiotic mice. *Genome Announc.* **2**, (2014).
2. Sarma-Rupavtarm, R. B., Ge, Z., Schauer, D. B., Fox, J. G. & Polz, M. F. Spatial distribution and stability of the eight microbial species of the altered schaedler flora in the mouse gastrointestinal tract. *Appl. Environ. Microbiol.* **70**, 2791–2800 (2004).
