# Supplementary material for: Spatial host–microbiome sequencing reveals niches in the mouse gut
Source: Nat Biotechnol. 2023 Nov 20;42(9):1394–403. doi: 10.1038/s41587-023-01988-1 (PMC11392810; doi:10.1038/s41587-023-01988-1)
Supplement: Supplementary file 2 — Reporting Summary [file 41587_2023_1988_MOESM2_ESM.pdf]

nature portfolio

Corresponding author(s): Sanja Vickovic and Aviv Regev

Last updated by author(s): Jul 24, 2023

Reporting Summary

Nature Portfolio wishes to improve the reproducibility of the work that we publish. This form provides structure for consistency and transparency in reporting. For further information on Nature Portfolio policies, see our Editorial Policies and the Editorial Policy Checklist.

Statistics

For all statistical analyses, confirm that the following items are present in the figure legend, table legend, main text, or Methods section.

n/a	Confirmed	
☐	☒	The exact sample size (*n*) for each experimental group/condition, given as a discrete number and unit of measurement
☐	☒	A statement on whether measurements were taken from distinct samples or whether the same sample was measured repeatedly
☐	☒	The statistical test(s) used AND whether they are one- or two-sided<br>*Only common tests should be described solely by name; describe more complex techniques in the Methods section.*
☐	☒	A description of all covariates tested
☐	☒	A description of any assumptions or corrections, such as tests of normality and adjustment for multiple comparisons
☐	☒	A full description of the statistical parameters including central tendency (e.g. means) or other basic estimates (e.g. regression coefficient) AND variation (e.g. standard deviation) or associated estimates of uncertainty (e.g. confidence intervals)
☐	☒	For null hypothesis testing, the test statistic (e.g. *F*, *t*, *r*) with confidence intervals, effect sizes, degrees of freedom and *P* value noted<br>*Give P values as exact values whenever suitable.*
☐	☒	For Bayesian analysis, information on the choice of priors and Markov chain Monte Carlo settings
☐	☒	For hierarchical and complex designs, identification of the appropriate level for tests and full reporting of outcomes
☐	☒	Estimates of effect sizes (e.g. Cohen's *d*, Pearson's *r*), indicating how they were calculated

Our web collection on statistics for biologists contains articles on many of the points above.

Software and code

Policy information about availability of computer code

Data collection	Image stitching was done using Vslide (v.1.0.0) with 60µm overlap and linear blending between fields of views. Images were extracted using jpg compression. Pooled libraries were sequenced with 25nt in the forward read and 55nt and 150nt in the reverse read on NextSeq and MiSeq (Illumina), respectively.
Data analysis	Fastq reads were generated with bcl2fastq2 (v.2.20.0) and trimmed to remove adaptor sequences using BBDuk (v.38.33). Trimmed reads were quality filtered using the same quality filtering step as in ST pipeline (v.1.7.6) but only reads longer than 100nt were kept. TagGD (v0.3.6) was used to connect the spatial barcode to each forward read (k-mer 6, mismatches 2, hamming distance clustering algorithm) and BWA mem (v0.7.17) with reference mouse genome (GRCm39) was used to remove host mapping sequences. Remaining reverse reads were mapped to the mouse gut bacterial reference (created as described in "Generation of mouse gut bacterial reference") using Kraken2 (v.2.0.9) (confidence 0.01). Reads originated from GF and SPF mice were mapped to the mouse gut bacterial reference, while reads originated from ASF mice were mapped to the ASF reference. Taxonomy assignments made by Kraken2 were improved using the deep learning model. UMIs with identical spatial barcodes and taxonomical assignments were collapsed using UMI-tools (v.1.0.0) resulting in a bacteria-by-barcode matrix. H&E images were processed using SpoTteR (https://github.com/klarman-cell-observatory/SpoTteR) where centroid coordinates were used as probable grid points and a rectangular grid was then fitted to these probable points using a local optimizer (nlminb, v.3.6.3). Splotch (v1.0) (https://github.com/tare/Splotch) is a hierarchical probabilistic that captures variation in ST data through modeling of different study design covariates such as individual's age or mouse condition, and was used for statistical analysis of spatial data. All custom code is available at: XXX

For manuscripts utilizing custom algorithms or software that are central to the research but not yet described in published literature, software must be made available to editors and reviewers. We strongly encourage code deposition in a community repository (e.g. GitHub). See the Nature Portfolio guidelines for submitting code & software for further information.

Data

Policy information about availability of data

All manuscripts must include a data availability statement. This statement should provide the following information, where applicable:

- Accession codes, unique identifiers, or web links for publicly available datasets
- A description of any restrictions on data availability
- For clinical datasets or third party data, please ensure that the statement adheres to our policy

All data have been deposited in the Single Cell Portal under accession SCP1447 (https://singlecell.broadinstitute.org/single_cell/study/SCP1447).

Human research participants

Policy information about studies involving human research participants and Sex and Gender in Research.

Reporting on sex and gender	N/A
Population characteristics	N/A
Recruitment	N/A
Ethics oversight	N/A

Note that full information on the approval of the study protocol must also be provided in the manuscript.

Field-specific reporting

Please select the one below that is the best fit for your research. If you are not sure, read the appropriate sections before making your selection.

☒ Life sciences ☐ Behavioural & social sciences ☐ Ecological, evolutionary & environmental sciences

For a reference copy of the document with all sections, see nature.com/documents/nr-reporting-summary-flat.pdf

Life sciences study design

All studies must disclose on these points even when the disclosure is negative.

Sample size	The number of biological and technical replicates was chosen based on preliminary experiments, so as to provide sufficient power for comparison. In each condition reported in the study, we used at least 3 independent mouse tissue sections to describe that condition.
Data exclusions	No data was exuded from the study.
Replication	In total, we applied SHM-seq to 124 tissue sections and collected data from 15,321 spatial spots (covered by the tissue) across the three conditions. All attempts at replication were successful.
Randomization	Animals were randomly distributed into cages and ear-punched by animal care staff. Cages of animals were randomly chosen for all experiments.
Blinding	Blinding was not relevant in this study because our experiments did not involve any human subjects and all data collectection, processing and analysis methods were quantitative and identical across all experimental groups.

Reporting for specific materials, systems and methods

We require information from authors about some types of materials, experimental systems and methods used in many studies. Here, indicate whether each material, system or method listed is relevant to your study. If you are not sure if a list item applies to your research, read the appropriate section before selecting a response.

Materials & experimental systems

n/a	Involved in the study
☒	☐ Antibodies
☒	☐ Eukaryotic cell lines
☒	☐ Palaeontology and archaeology
☐	☒ Animals and other organisms
☒	☐ Clinical data
☒	☐ Dual use research of concern

Methods

n/a	Involved in the study
☒	☐ ChIP-seq
☒	☐ Flow cytometry
☒	☐ MRI-based neuroimaging

Animals and other research organisms

Policy information about studies involving animals; ARRIVE guidelines recommended for reporting animal research, and Sex and Gender in Research

Laboratory animals	Adult (>6 weeks of age) C57BL/6 specific pathogen-free mice (SPF), Adult C57BL/6 germ-free mice (GF) and Adult C57BL/6 germ-free mice that have been colonized with ASF. Adult C57BL/6 germ-free mice (denoted GF) were obtained from Taconic Biosciences (USA) and maintained in a gnotobiotic environment. Some of these mice were randomly selected and inoculated with ASF31 over several generations and used when >6 weeks of age. After colonization, ASF mice (denoted ASF) were housed in sterile conditions, and tested with PCR to ensure sterility was maintained. Animal housing room temperatures are monitories and maintained at all times according to species-specific needs. Humidity is maintained at 30-70%. Light intensity and light cycle timing were carefuly regulated by Broad Institute animal facilities. Automated light timers ensured a consistent light&dark cycle.
Wild animals	No wild animals were used in this study.
Reporting on sex	N/A
Field-collected samples	No samples were collected in the field.
Ethics oversight	Adult C57BL/6 specific pathogen-free mice (SPF) were purchased from The Jackson Laboratory (Bar Harbor, ME) and maintained in accordance with ethical guidelines monitored by the Institutional Animal Care and Use Committees (IACUC) established by the Division of Comparative Medicine at the Broad Institute of MIT and Harvard, and consistent with the Guide for Care and Use of Laboratory Animals, National Research Council, 1996 (institutional animal welfare assurance no. A4711-01), with protocol 0122-10-16.

Note that full information on the approval of the study protocol must also be provided in the manuscript.
